# Evidence Mapping of ctDNA Reporting in Pancreatic Ductal Adenocarcinoma: Toward a Shared Quantitative Language for ctDNA

**DOI:** 10.3390/cancers18081318

**Published:** 2026-04-21

**Authors:** Daniel Croagh, Saeed Aslani

**Affiliations:** Department of Surgery, School of Clinical Sciences Monash Health, Monash University, Melbourne, VIC 3168, Australia

**Keywords:** pancreatic cancer, KRAS, molecular diagnostic techniques, circulating tumour DNA, digital droplet PCR, high-throughput nucleotide sequencing, reference standards

## Abstract

**Background/Objectives**: Circulating tumour DNA (ctDNA) assays enable non-invasive assessment of tumour burden and treatment response in oncology. However, quantitative ctDNA outputs (such as variant allele frequency, tumour fraction, and aggregate burden scores) remain difficult to interpret and compare across platforms. This evidence-mapping review evaluates current quantitative reporting approaches in pancreatic ductal adenocarcinoma (PDAC) and examines the potential role of KRAS mutant ctDNA as a biologically grounded reference metric. **Methods**: A systematic literature search was conducted across PubMed/MEDLINE and Scopus to identify studies reporting quantitative ctDNA metrics in PDAC. Eligible studies included those measuring plasma KRAS mutations and/or reporting variant allele frequency, tumour fraction, or multi-locus aggregate metrics. Additional relevant primary studies identified through broader manual searching of PubMed were assessed against the same prespecified eligibility and classification criteria before inclusion. Data were synthesised narratively, focusing on reporting frameworks, units of measurement, assay characteristics, and the interpretability of quantitative outputs across platforms. **Results**: Substantial heterogeneity was observed in ctDNA quantification methods and reporting standards. Ratio-based metrics such as variant allele frequency and tumour fraction were commonly used but varied according to assay design, plasma input volume, and background cell-free DNA levels. Few studies reported absolute mutant molecule counts per unit volume. Given that approximately 90–95% of PDACs harbour truncal activating KRAS mutations, plasma KRAS was consistently represented across platforms and demonstrated potential as a shared quantitative anchor. Limited standardisation was noted in distinguishing detectability from quantifiability based on sampling depth and counting statistics. **Conclusions**: Current ctDNA reporting in PDAC lacks a shared quantitative reference, limiting cross-study comparability. Reporting KRAS mutant molecules per millilitre and adopting an assay-agnostic framework distinguishing detection from quantification may improve interpretability, support harmonisation across platforms, and facilitate cumulative learning in pancreatic cancer ctDNA research.

## Simple Summary

Blood tests that measure fragments of tumour DNA are increasingly used to monitor cancer without the need for repeated biopsies. However, the results of these tests are often difficult to compare because different laboratories report them using different measurements that do not always clearly reflect how much cancer is present. In pancreatic cancer, most tumours share the same key genetic change in the *KRAS* gene. This creates an opportunity to use this mutation as a common reference point. We suggest that, instead of relying on percentage-based measurements, researchers should report the actual number of mutant KRAS DNA molecules in a defined volume of blood. We also propose a simple framework to distinguish between merely detecting tumour DNA and accurately measuring it. This approach could improve consistency across studies and help the research community interpret and compare results more reliably. This could help align liquid biopsy reports from different laboratories, making them easier for clinicians to interpret in the same way.

## 1. Introduction

Circulating tumour DNA (ctDNA) assays have substantially expanded what can be measured from blood in oncology [1,2,3,4,5]. Contemporary platforms now report a wide range of quantitative outputs, including variant allele frequency (VAF), tumour fraction, composite allele frequency metrics, and absolute measures such as mutant molecules per millilitre.

Despite this apparent quantitative sophistication, the biological meaning of ctDNA measurements remains difficult to interpret, particularly when results are compared across assays, studies, or platforms. Different reporting conventions reflect different implicit assumptions about background cfDNA, variant selection, and signal aggregation. As a result, ctDNA evidence often accumulates in parallel rather than cumulatively, limiting cross-study synthesis and clinical translation [6].

This challenge does not arise from a lack of analytical capability. Rather, it reflects the absence of a shared quantitative reference that clarifies what reported ctDNA values represent biologically. Without such a reference, both ratio-based metrics (such as VAF) and complex aggregated metrics remain opaque and this limits our understanding of how they relate to the actual burden of tumour-derived DNA in the circulation and potentially constrains their clinical adoption.

### 1.1. Why Quantitative Molecular Frameworks Are Straightforward in Virology but Difficult in Oncology

Quantitative molecular assays have been highly successful in fields such as virology. In viral infections, such as hepatitis B, the target genome is uniform, the biological source of circulating nucleic acid is well-defined, and viral load directly reflects pathogen burden and replication. As a result, molecular measurements can be standardised, benchmarked, and expressed in clinically interpretable units that are consistent across platforms and studies [7,8,9,10,11].

In oncology, by contrast, ctDNA originates from a heterogeneous and evolving tumour population that is largely unique to each patient. It is also diluted by variable host-derived cell-free DNA, and reflects complex biological processes including cell death, treatment response, and tumour microenvironment dynamics. The biological source of circulating DNA is therefore neither uniform nor stable. This makes it inherently more difficult to define quantitative ctDNA units that can be interpreted consistently across patients, platforms, and disease states [12,13,14].

### 1.2. Early ctDNA Applications: Single-Locus Assays and the Rise in VAF

Early ctDNA applications in oncology benefited from conceptual simplicity by focusing on single, well-defined tumour mutations [15,16]. Measuring a single truncal alteration offered a direct and interpretable signal of tumour-derived DNA in circulation, particularly in settings where ctDNA shedding was high and mutant molecules were consistently detectable above the limit of quantification.

In these early single-locus assays, VAF became the dominant reporting metric [17,18]. This reflected technical convenience and historical precedent: VAF is the native output of most sequencing and digital PCR workflows, normalises for variable DNA input, and had long been used in tissue genomics to describe clonal architecture. In plasma-based assays, VAF therefore provided a familiar, platform-agnostic way to express mutant signal relative to background cfDNA.

However, it is not strictly quantitative and the use of VAF in circulating blood masked an important biological assumption, that the background cfDNA pool is relatively stable. In reality, normal-tissue apoptosis, inflammation, and treatment-related injury can substantially alter the denominator of the VAF calculation, independent of tumour dynamics [19]. Despite these recognised limitations, VAF remained widely used because it was analytically convenient, historically entrenched, and easy to standardise across platforms.

### 1.3. The Shift to Multi-Locus Assays and the Loss of a Common Benchmark

In many solid tumours, including pancreatic cancer, ctDNA shedding is often low, particularly during effective therapy or in minimal residual disease states. At these low concentrations, single-locus measurements become dominated by Poisson sampling effects, stochastic dropout, and assay limits of quantification [15,16]. Under such conditions, absence of detection may reflect sampling noise rather than true biological absence.

To recover sensitivity and improve signal stability, assays increasingly aggregated information across multiple tumour-derived variants [17,18,20]. This drove the widespread adoption of multi-locus ctDNA platforms, which sum signal across truncal and subclonal mutations to improve detection in low-shedding contexts.

However, this gain in sensitivity came at the cost of interpretive simplicity. Multi-locus assays rely on complex analytical pipelines that differ in how variants are selected, weighted, filtered, and modelled. These pipelines are often only partially disclosed, and their aggregate quantitative outputs—such as tumour fraction, composite allele frequency metrics, or proprietary burden scores—operate on implicitly different scales. As a result, cross-platform benchmarking became difficult, and quantitative ctDNA results increasingly accumulated in parallel rather than cumulatively [17,18,19,20].

The purpose of this review is to evaluate how ctDNA tumour burden is currently quantified in pancreatic cancer and to assess whether greater metrological rigour could improve interpretability, interoperability, and cumulative evidence synthesis.

## 2. Materials and Methods

### 2.1. Study Design

This study was conducted as an evidence-mapping review and reported using PRISMA (Preferred Reporting Items for Systematic Reviews and Meta-Analyses) guidelines [21] (Supplementary File S1). The review was undertaken with an evidence-mapping objective, focusing on how ctDNA reporting approaches are classified and interpreted rather than on pooling clinical effect sizes. The review was not prospectively registered.

The analysis focused on identifying, classifying, and synthesising ctDNA reporting conventions and quantitative metrics used across studies. The primary objective was to evaluate whether current reporting practices support biologically interpretable and interoperable ctDNA quantification.

The analysis comprised three complementary components: single-locus KRAS ctDNA studies, multi-locus NGS ctDNA assays, and published systematic reviews and meta-analyses of ctDNA in PDAC. These components were analysed in parallel to assess reporting practices across the evidence pipeline, from primary assay outputs to secondary evidence synthesis. Study identification was based on structured database searches, supplemented by targeted manual searching of reference lists and relevant articles to ensure completeness, with all records assessed using the same predefined eligibility and classification framework.

### 2.2. Literature Search Strategy

A structured literature search was performed in PubMed/MEDLINE and Scopus from database inception to 30 March 2026. Separate search strategies were constructed for each study component. The complete electronic search strategies for PubMed/MEDLINE and Scopus, including Boolean operators, field tags, and limits used, are provided in Supplementary File S2.

Searches targeting single-locus studies combined terms related to PDAC, KRAS, circulating tumour DNA, and digital PCR technologies. Searches targeting multi-locus assays combined PDAC and ctDNA terms with keywords related to NGS, gene panels, tumour fraction, and liquid biopsy. Searches targeting meta-analyses combined PDAC and ctDNA terms with descriptors of systematic review and meta-analysis methodology.

### 2.3. Study Selection

Duplicate records were removed before title and abstract screening. Title/abstract screening, full-text eligibility assessment and final study classification were reviewed by both authors (D.C. and S.A.), with disagreements resolved by consensus. Records excluded at the title/abstract stage were removed for lack of relevance to PDAC ctDNA, including non-PDAC studies, non-ctDNA studies, non-human studies, review articles, and records without relevant ctDNA reporting.

Studies were included if they involved human PDAC cohorts, analysed plasma-derived ctDNA, and reported ctDNA using either single-locus KRAS assays, multi-locus NGS-based platforms, or systematic synthesis of ctDNA data. Studies were excluded if they were limited to tissue-based sequencing, non-ctDNA biomarkers, or purely technical assay development without clinically relevant ctDNA reporting. Studies were also excluded if ctDNA/cfDNA was not the principal analyte or reporting framework, including multianalyte biomarker panels in which cfDNA/ctDNA formed only one component of a broader composite assay. Narrative reviews without systematic synthesis were excluded from the meta-analysis component. Additional non-eligible publication types, including case reports, conference abstracts without a corresponding full-text primary publication, book chapters, editorials, and letters, were excluded.

For primary studies, inclusion required reporting of ctDNA measurement at the assay level. Studies were classified into three mutually exclusive reporting paradigms: binary, relative, and absolute. Classification was based on the dominant ctDNA metric reported in each study, rather than the downstream clinical use of that metric. Binary classification was assigned to studies that used ctDNA as a dichotomous variable, such as detected versus not detected or MRD positive versus negative. Relative classification was assigned to studies reporting ctDNA as a proportion, ratio, or assay-derived burden metric, such as variant allele fraction, mutant allele fraction, circulating mutational allele fraction, allele fraction, tumour fraction, ctDNA burden, or mutation counts normalised to genome equivalents rather than plasma volume. Absolute classification was reserved for studies reporting ctDNA concentrations in directly interpretable plasma volume-normalised units, such as mutant copies or mutant molecules per mL of plasma. Where studies reported more than one form of quantification, classification was determined by the dominant analytical framework used in the study.

### 2.4. Data Extraction

Data extraction was initially performed by one reviewer (S.A.) using predefined templates tailored to each study category. Extracted data and study classification were then reviewed by both authors (D.C. and S.A.), and any discrepancies were resolved by consensus. 

For single-locus KRAS ctDNA studies, information was extracted on assay type, reported ctDNA metric, and clinical application. Extracted ctDNA metrics were recorded in their original form (such as detected/not detected, VAF, MAF, allele fraction, ctDNA burden, or copies/mL), and each study was then assigned to a single evidence-mapping category (binary, relative, or absolute) according to its dominant analytical framework.

For multi-locus ctDNA studies, extracted variables included the sequencing platform, reported ctDNA metric, inclusion and explicit quantification of KRAS, and the intended clinical application. In addition, we recorded whether the study explicitly reported three predefined methodological elements relevant to interpretability: (i) variant selection rules, (ii) signal aggregation or composite-score rules, and (iii) modelling or calibration assumptions used to derive the reported ctDNA metric. These items were extracted descriptively and were not used as a formal quality score or evaluative ranking.

For systematic reviews and meta-analyses, extracted variables included clinical endpoints, the type of ctDNA data synthesised, and whether quantitative calibration across assays or absolute ctDNA thresholds were proposed.

### 2.5. Data Synthesis

Data synthesis was descriptive and narrative. Reporting practices were summarised using counts and proportions, and patterns in metric usage were interpreted qualitatively across study categories.

No pooled statistical meta-analysis was performed, as the primary aim was to evaluate the comparability and interpretability of quantitative ctDNA reporting rather than to estimate summary effect sizes. Given the methodological focus of this evidence-mapping review, formal risk-of-bias assessment was not undertaken. Instead, studies were evaluated according to reporting characteristics relevant to quantitative interpretability, including metric type, plasma volume normalisation, reporting of detection or quantification limits, and analytical transparency.

### 2.6. Interpretive Framework

Findings were interpreted in the context of a biologically grounded quantitative framework for ctDNA reporting in PDAC, centred on reporting mutant molecule counts normalised to plasma volume with explicit acknowledgment of LOQ. This framework was not imposed during study selection or data extraction but was used to contextualise current reporting practices.

## 3. Results

**Figure 1:**
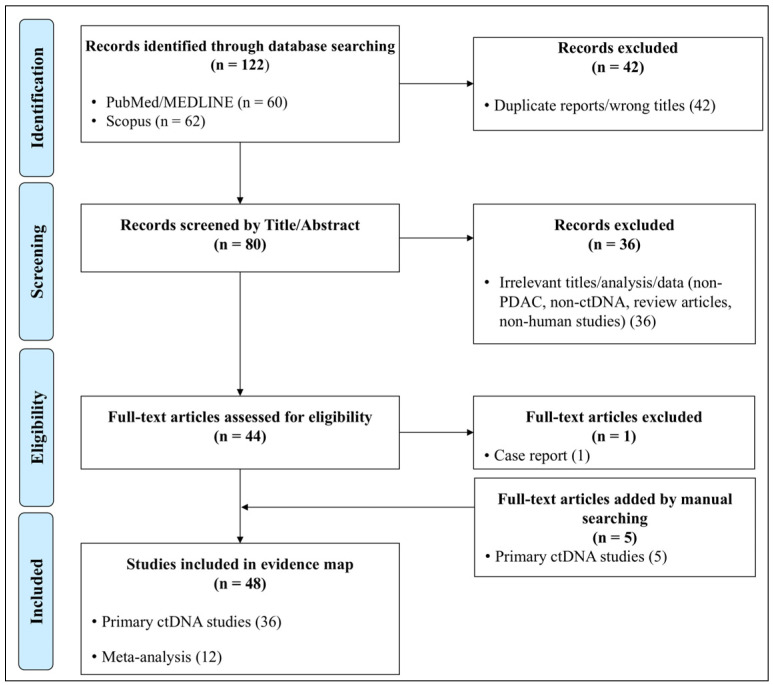
Flow diagram of database yields, duplicate removal, title/abstract screening, full-text eligibility assessment, and inclusion of primary ctDNA studies and published systematic reviews/meta-analyses in the evidence-mapping review, based on PRISMA guidelines.

The database search yielded 122 records in total, comprising 60 records from PubMed/MEDLINE and 62 records from Scopus. After removal of 42 duplicate records, 80 unique records remained for title and abstract screening. Following title/abstract screening, 36 records were excluded and 44 full-text articles were assessed for eligibility. After full-text review, one record was excluded from the final evidence map as it was a case report involving only one patient. An additional five studies were identified through manual searching. Ultimately, 48 studies were included in the evidence map, comprising 36 primary ctDNA studies and 12 meta-analysis studies. The full study selection workflow, including database-specific yields, duplicate removal, title/abstract screening, full-text assessment, and final inclusion, is summarised in Figure 1.

### 3.1. Quantification Approaches in Primary ctDNA Studies of Pancreatic Cancer

Thirty-six primary ctDNA studies in pancreatic cancer were included in this evidence-mapping review, encompassing both single-locus KRAS assays and multi-locus panel-based approaches [21,22,23,24,25,26,27,28,29,30,31,32,33,34,35,36,37,38,39,40,41,42,43,44,45,46,47,48,49,50,51,52,53,54,55,56] (Figure 1; Table 1). For evidence-mapping purposes, each primary study was assigned to a single dominant reporting category (binary, relative quantitative, or absolute quantitative) according to the principal ctDNA metric reported in the study. Using the predefined mutually exclusive classification rules, the 36 primary studies comprised 12 binary studies, 21 relative studies, and 3 absolute studies. We acknowledge that the boundary between binary and relative classification is not always sharp and may involve interpretive judgement in studies reporting more than one ctDNA metric. However, this does not alter the central observation that absolute plasma volume-normalised reporting remained uncommon across the included literature. Collectively, these studies span the clinical spectrum of pancreatic cancer, including early-stage, resectable, borderline resectable, locally advanced, metastatic, and minimal residual disease (MRD) settings, and represent a substantial cumulative sample size.

Despite this breadth of biological coverage and increasing technical sophistication over time, marked heterogeneity was observed in how ctDNA burden was reported. As summarised in Table 1, the majority of studies relied on either binary detection-based output [21,22,23,24,25,26,27,28,29,30,31,32] or relative quantitative measures such as variant allele fraction (VAF), mutant allele fraction (MAF), allele fraction, circulating mutational allele fraction, tumour fraction, dominant clone allele frequency, or ctDNA burden [33,34,35,36,37,38,39,40,41,42,43,44,45,46,47,48,49,50,51,52,56], whereas absolute volume-normalised quantification remained uncommon [53,54,55].

Binary reporting was particularly common in studies that used ctDNA primarily as a dichotomous variable, such as detectable versus not detected, mutation-positive versus mutation-negative, or MRD positive versus negative [21,22,23,24,25,26,27,28,29,30,31,32]. Relative quantitative approaches were more frequent overall and included VAF-based, MAF-based, tumour fraction-based, and related burden-oriented metrics [33,34,35,36,37,38,39,40,41,42,43,44,45,46,47,48,49,50,51,52,56]. In contrast, only three studies reported ctDNA using absolute quantitative units such as copies or mutant molecules per mL plasma [53,54,55].

This pattern was observed across both single-locus and multi-locus studies. Even in technically constrained KRAS-focused ddPCR assays, reporting varied between binary detection [21,22,26,28], relative mutant fraction measures [33,34,35,40,48,56], and less commonly absolute molecule-per-volume quantification [53,54,55]. In more recent multi-locus studies, reporting diversity increased further, with targeted NGS, tumour-informed approaches, and broader panel-based assays often prioritising sensitivity or classification over transferable quantitative outputs [25,27,29,31,32,36,37,41,42,43,44,45,46,47,49,50,51,52].

Notably, the persistence of non-absolute reporting was not limited to earlier studies. Across time and assay types, most studies continued to favour binary or relative paradigms rather than harmonised absolute quantification. Overall, these findings indicate that, despite substantial cumulative patient volume, broad disease representation, and advances in assay technology, ctDNA studies in pancreatic cancer remain dominated by non-harmonised reporting conventions. This limits direct comparison across studies, impairs cumulative interpretation of ctDNA burden, and supports the need for a more interoperable quantitative framework.

**Table 1:** Quantification approaches in ctDNA studies of pancreatic cancer. Despite increasing technical sophistication over time, most included studies reported ctDNA using binary detection or relative measures rather than absolute volume-normalised outputs, highlighting the persistent lack of a harmonised quantitative framework across single-locus and multi-locus assays in PDAC. Studies were assigned to one mutually exclusive reporting category according to the dominant reported ctDNA metric in the original study.

Study	Year	*n*	Clinical Setting	Technique	Locus Type	Paradigm	Metric
**Binary**							
Brychta et al. [21]	2016	50	Early-stage/diagnostic	Digital PCR	Single (KRAS)	Binary	Mutation detected
Chen et al. [22]	2017	189	Advanced/unresectable	ddPCR	Single (KRAS)	Binary	KRAS ctDNA per 10^5^ GEq
Guo et al. [23]	2020	113	Resectable	NGS	Single (KRAS)	Binary	KRAS G12D detected
Macgregor-Das et al. [24]	2020	67	Diagnostic	Digital NGS	Single (KRAS/GNAS)	Binary	Hotspot mutation detected
van der Sijde et al. [25]	2021	48	Mixed	NGS	Multi-locus	Binary	TP53 mutation detected
Wang et al. [26]	2022	105	Mixed/diagnostic	ddPCR	Single (KRAS)	Binary	Mutation detected
Watanabe et al. [27]	2022	145	Resected	Tumour-informed sequencing	Multi-locus	Binary	ctDNA detected
Hata et al. [28]	2023	66	Resected	ddPCR	Single (KRAS)	Binary	Detected vs. not detected
Levink et al. [29]	2023	26	Diagnostic	Sequencing	Multi-locus	Binary	Mutation detection
Lee et al. [30]	2024	128	Advanced	ddPCR/sequencing	Single (KRAS)	Binary	Mutation detected
Murakami et al. [31]	2025	135	Resectable/BRPC	Sequencing	Multi-locus	Binary	ctDNA detection
Zhang et al. [32]	2025	39	Mixed	Tumour-informed sequencing	Multi-locus	Binary	MRD positive/negative
**Relative**							
Kinugasa et al. [33]	2015	75	Mixed	ddPCR	Single (KRAS)	Relative	VAF
Takai et al. [34]	2015	259	Mixed	ddPCR + sequencing	Single (KRAS)	Relative	MAF
Tjensvoll et al. [35]	2016	14	Metastatic	ddPCR	Single (KRAS)	Relative	VAF dynamics
Berger et al. [36]	2017	20	Advanced	Sequencing	Multi-locus	Relative	CMAF
Pietrasz et al. [37]	2017	135	Mixed	NGS + ddPCR	Multi-locus	Relative	VAF
Kruger et al. [38]	2018	54	Advanced	BEAMing	Single (KRAS)	Relative	Mutation/ng
Perets et al. [39]	2018	17	Metastatic	NGS	Single (KRAS)	Relative	Mutant KRAS ctDNA level
Wang et al. [40]	2019	110	Mixed	ddPCR	Single (KRAS)	Relative	MAF
Wei et al. [41]	2019	38	Advanced	NGS panel	Multi-locus	Relative	MAF
Strijker et al. [42]	2020	60	Metastatic	Custom NGS panel	Multi-locus	Relative	ctDNA quantity/highest VAF
Wei et al. [43]	2020	70	Advanced/metastatic	Shallow WGS	Multi-locus	Relative	Tumour fraction (TFx)
Takano et al. [44]	2021	24	Mixed/diagnostic	Digital NGS	Multi-locus	Relative	MAF
Botrus et al. [45]	2022	104	Advanced	NGS (Guardant)	Multi-locus	Relative	DCAF/thresholded VAF
Lee et al. [46]	2022	70	Resectable	NGS	Multi-locus	Relative	VAF/hGE/mL
Sellahewa et al. [56]	2023	81	Mixed	ddPCR	Single (KRAS)	Relative	Thresholded VAF
Lapin et al. [47]	2023	56	Advanced	Targeted sequencing	Multi-locus	Relative	ctDNA burden
Tanaka et al. [48]	2023	46	Mixed	ddPCR + melting curve	Single (KRAS)	Relative	VAF
Huerta et al. [49]	2024	80 *	Advanced	Whole exome sequencing	Single (KRAS)	Relative	VAF
Theparee et al. [50]	2024	81 †	Mixed	Sequencing	Multi-locus	Relative	Allele fraction
Petersson et al. [51]	2025	60	Newly diagnosed	Targeted ctDNA assay	Multi-locus	Relative	ctDNA burden
Zavrtanik Čarni et al. [52]	2025	50	Resected	PCR-based	Multi-locus	Relative	VAF
**Absolute**							
Hadano et al. [53]	2016	105	Resected	ddPCR	Single (KRAS)	Absolute	KRAS copies/mL
Lin et al. [54]	2018	65	Locally advanced/IRE	ddPCR	Single (KRAS)	Absolute	KRAS mutations/mL
Hussung et al. [55]	2024	47	Stage IV	ddPCR	Multi-locus	Absolute	Mutant copies/mL

**Abbreviations**: BRPC, borderline resectable pancreatic cancer; CMAF, circulating mutational allele fraction; ctDNA, circulating tumour DNA; ddPCR, droplet digital polymerase chain reaction; KRAS, Kirsten rat sarcoma viral oncogene homologue; MRD, minimal residual disease; NGS, next-generation sequencing; PDAC, pancreatic ductal adenocarcinoma; PCR, polymerase chain reaction; VAF, variant allele fraction. **Footnotes**: * For Huerta et al. [49], the overall cohort comprised 80 metastatic PDAC patients; paired tumour-plasma whole-exome sequencing was performed in a discovery subset. † For Theparee et al. [50], 95 plasma samples were collected, but 81 patients had adequate cfDNA for sequencing and were included in the analytic cohort.

### 3.2. Evidence from Published Meta-Analyses of ctDNA in PDAC

Twelve published systematic reviews and meta-analyses evaluating ctDNA in PDAC were included [57,58,59,60,61,62,63,64,65,66,67,68]. Meta-analyses were treated as a separate evidence stream and were not cross-classified with the primary study reporting categories shown in Table 1. Across all meta-analyses, quantitative synthesis was performed using categorical or relative measures, rather than absolute ctDNA burden (Table 2).

Most meta-analyses pooled hazard ratios for overall survival, progression-free survival, or recurrence-free survival, comparing ctDNA-positive versus ctDNA-negative patients or “high” versus “low” ctDNA groups defined using study-specific thresholds [57,58,59,60,61,62,63,65,67,68]. In addition, one broader cfDNA-focused meta-analysis pooled prognostic hazard ratios across multiple cfDNA-related variables, including mutation status, ctDNA presence, hypermethylation, and higher cfDNA concentration, rather than using a single harmonised quantitative ctDNA metric [68]. Diagnostic meta-analyses similarly pooled sensitivity, specificity, diagnostic odds ratios, or area under the curve values [64,66]. A recent surgery-focused meta-analysis specifically pooled preoperative and postoperative ctDNA status against disease-free survival and overall survival in resected PDAC, including subgroup analyses for upfront surgery and neoadjuvant treatment cohorts [67]. Importantly, none of the meta-analyses attempted or enabled quantitative calibration across assays or platforms, such as harmonisation between VAF-based and molecule-based reporting.

Where terms such as “high ctDNA level” were used, thresholds were typically derived within individual studies and were not externally validated or transferable [57,59,61,67]. No meta-analysis proposed absolute ctDNA concentration cut-offs or a unified quantitative framework that would allow cross-study or cross-platform synthesis of tumour burden.

## 4. Discussion

Pancreatic ductal adenocarcinoma (PDAC) is subject to the same quantitative challenges that affect ctDNA interpretation across solid tumours. VAF is biologically unstable in circulating blood, and ctDNA shedding is often low—particularly during effective therapy or in minimal residual disease states—limiting the reliability of single-locus measurements at low molecule counts. In contrast, multi-locus assays rely on heterogeneous analytical pipelines whose quantitative outputs are difficult to compare across platforms [69,70,71].

The structured evidence-mapping of the pancreatic cancer ctDNA literature supports this interpretation. Across the included primary studies, binary and relative ctDNA reporting paradigms predominated, whereas absolute volume-normalised quantification was uncommon (Table 1). This pattern persisted even in more recent studies and appeared particularly influenced by the rise in multi-locus and MRD-oriented assays, which often prioritised detection sensitivity or proprietary composite classification over transferable absolute burden reporting.

These constraints have shaped how ctDNA has been operationalised in pancreatic cancer. Because most assays do not report tumour-derived DNA on a stable quantitative scale, clinical applications largely focused on binary questions such as prognostic stratification, molecular relapse detection, or MRD classification [36,42,72,73,74,75]. While this approach has proven clinically useful, binary MRD frameworks collapse biologically distinct levels of ctDNA signal into a single positive category.

Even within this apparently binary setting, however, absolute quantitation allows additional biological information to be recovered. Under an absolute molecules-per-millilitre reporting framework, a negative result would remain unchanged, but positive results could be expressed in quantitative terms, distinguishing between low-level detectable ctDNA and clearly quantifiable ctDNA burden. A shared quantitative reporting language would therefore allow MRD states to be stratified, for example by separating MRD-low (low-level detectable ctDNA) from MRD-high (clearly quantifiable ctDNA burden).

Such stratification may have distinct biological and clinical implications. MRD-low may reflect minimal tumour DNA release or transient shedding, whereas MRD-high may represent established residual disease associated with greater recurrence risk. This framework therefore creates a path for testing absolute KRAS molecules per millilitre as graded MRD thresholds or molecular R1-style markers in prospective adjuvant therapy studies.

More broadly, the dominance of binary or relative ctDNA metrics means that ctDNA in PDAC has largely functioned as a prognostic classifier rather than a quantitative biomarker capable of supporting calibrated treatment monitoring. Relative measures such as VAF, tumour fraction, or binary detection status can still provide clinically useful information, particularly for prognostic stratification. However, because these metrics lack a stable biological reference, they provide limited insight into the absolute burden of tumour-derived DNA in the circulation. These observations suggest that the central challenge in pancreatic cancer is not the absence of a meaningful ctDNA signal, but the absence of a stable biological reference against which that signal can be quantitatively interpreted.

### 4.1. Why Pancreatic Cancer Offers a Unique Opportunity

Despite sharing these challenges, PDAC differs from most solid tumours in one critical respect: approximately 90–95% of cases harbour activating KRAS mutations, which are typically truncal and present in all cancer cells [76,77,78,79]. As a result, KRAS is included—explicitly or implicitly—in most ctDNA assays applied to pancreatic cancer, ranging from single-locus digital PCR to broad multi-locus sequencing panels.

When detectable above the limit of quantification, plasma KRAS provides a biologically grounded reference signal that reflects tumour-derived DNA entering the circulation. While KRAS is not a perfect surrogate for tumour burden, its ubiquity and truncal nature make it uniquely suited to serve as a common quantitative reference in this disease.

This near-universal inclusion of KRAS creates an opportunity that does not exist in most other tumour types: a shared biological signal can be extracted from otherwise heterogeneous ctDNA platforms without altering their analytical pipelines.

### 4.2. KRAS as a Dual-Solution Calibration Axis in PDAC

Reporting KRAS mutant molecules per millilitre resolves two distinct quantitative problems in pancreatic cancer ctDNA analysis:

1. In single-locus assays, which predominantly target KRAS, absolute molecule counts replace unstable ratio-based VAF with a biologically interpretable burden metric.
2. In multi-locus assays, where KRAS is almost universally included as a truncal founder mutation, explicit KRAS quantification provides a shared calibration axis that allows aggregate ctDNA metrics to be interpreted on a common biological scale.

In this framework, KRAS is not privileged because it is the best biomarker, but because its ubiquity enables a shared quantitative language across assay types.

### 4.3. Proposed Minimal Reporting and Calibration Framework for ctDNA in PDAC

To further improve interpretability and enable meaningful cross-study comparison, we propose a minimal, assay-agnostic reporting standard for quantitative ctDNA results. For each sample, studies should report:

1. The total number of mutant molecules detected in the assay run (*k*);
2. The plasma-equivalent volume represented in that run (*V*, mL);
3. The derived concentration (*k*/*V*, mutant molecules per millilitre).

In count-based platforms such as droplet digital PCR, measurement precision is fundamentally limited by Poisson sampling noise, such that the theoretical lower bound on relative imprecision (coefficient of variation, CV) is approximately 1/√k. As a result, relative imprecision increases rapidly at low molecule counts. While this approximation is informative for direct counting processes, it does not by itself define the variance structure of multi-locus ctDNA outputs, where uncertainty may also arise from library preparation efficiency, molecular tagging, background error suppression, locus selection, panel breadth, signal aggregation rules, and downstream filtering or modelling assumptions.

In contrast, multi-locus ctDNA assays may achieve low relative imprecision through the aggregation of signal across multiple variants, even when individual loci such as KRAS fall below the limit of quantification. In these settings, the lack of quantifiable KRAS does not imply poor analytical precision. We therefore suggest that, where available, derived measures of quantitative uncertainty (such as CV or equivalent assay-specific metrics) be explicitly reported alongside absolute ctDNA estimates. This allows confidence in quantitative interpretation to be assessed independently of KRAS detectability, while preserving the role of KRAS as a biological calibration reference when measurable.

On this basis, we suggest classifying results as:

• Quantifiable: ≥10 mutant molecules per run (theoretical Poisson CV ≤ ~32%);
• Detectable but below the limit of quantification: 1–9 molecules;
• Not detected: 0 molecules.

This classification is intended as a theoretical counting framework for direct single-locus molecule counting assays and should not be interpreted as an established cross-platform standard for aggregate multi-locus outputs. These count-based categories are proposed as a theoretical reporting framework derived from Poisson sampling behaviour and are intended to improve interpretability; they should not be regarded as clinically validated prognostic or treatment-decision thresholds in PDAC at the present time. This framework makes measurement uncertainty explicit, accommodates variable plasma input volumes, and allows ctDNA results—particularly KRAS measurements in PDAC—to be interpreted on a biologically meaningful and statistically coherent scale across platforms and studies, without reliance on ratio-based metrics whose denominators may vary independently of tumour burden. Prospective studies will be required to determine whether the proposed boundaries between detectable but not quantifiable and quantifiable KRAS levels have discriminative value for prognostic stratification, postoperative MRD assessment, or dynamic treatment monitoring. Accordingly, the thresholds discussed here should be understood as a conceptual interpretive framework anchored in direct molecule counting, not as a validated quantitative standard for all ctDNA platforms.

### 4.4. A Pragmatic Anchoring Strategy Using KRAS in PDAC

In pancreatic cancer, this framework can be applied without altering assay algorithms or imposing new standards. When plasma KRAS is detectable above a defined limit of quantification, reporting KRAS mutant molecule counts alongside the plasma-equivalent input volume allows concentrations to be interpreted with explicit reference to measurement precision.

When such count-based reporting is provided in parallel with aggregate ctDNA metrics, KRAS serves as an external biological anchor against which those metrics can be interpreted. Where KRAS subsequently falls below the limit of quantification—such as during effective therapy—prior calibration allows ongoing aggregate ctDNA measurements to be interpreted in context.

Many aggregate ctDNA metrics are already interpreted, implicitly, as reflecting tumour-derived DNA burden. In pancreatic cancer, where KRAS is near-universal and truncal, this interpretation is commonly understood as relating to the dominant KRAS signal, even when KRAS itself is not directly quantifiable. The present framework does not redefine what these assays measure; rather, it makes explicit the biological coordinate system in which such measurements are already being interpreted. In this context, KRAS-equivalent molecules per millilitre represent a transparent description of an inferred quantity that many platforms already approximate, conditional on calibration to directly measurable KRAS when available. A practical calibration example is as follows. At a timepoint where KRAS is directly quantifiable, suppose a sample contains 20 measured KRAS mutant molecules from 4 mL plasma, equivalent to 5 KRAS molecules/mL, while the multi-locus assay detects 40 aggregate mutant molecules across all tracked variants in the same plasma-equivalent volume. In that sample, the calibration factor is therefore 20/40 = 0.5 KRAS-equivalent molecules per aggregate mutant molecule. If, at a later timepoint, KRAS itself is no longer quantifiable but the multi-locus assay detects 12 aggregate mutant molecules from the same 4 mL plasma-equivalent input, the inferred burden would be 12 × 0.5 = 6 KRAS-equivalent molecules in the assay run, corresponding to 1.5 KRAS-equivalent molecules/mL. This does not imply direct measurement of KRAS below the LOQ; rather, it expresses aggregate ctDNA signal on a KRAS-anchored biological scale established during prior co-measurement. Whether KRAS-equivalent molecules/mL remain linearly related to aggregate mutant signal below the limit of quantification for single-locus KRAS measurement has not been established. Accordingly, the calibration example should be understood as an illustrative conceptual framework rather than an empirically validated relationship across the full low-abundance range. This question could be explored in future studies using simultaneous portal and systemic venous sampling, particularly if portal blood proves enriched for ctDNA relative to systemic blood because of reduced first-pass hepatic clearance and less systemic dilution.

Importantly, this approach does not require assumptions about tumour cell counts, does not mandate how assays perform aggregation, and does not impose platform-specific analytical standards. Practical adoption will still require several implementation steps. First, assays would need to report mutant KRAS molecule counts together with the plasma-equivalent volume represented in the assay run, which is not yet routine in many reports. Second, commercial and proprietary platforms would need to expose sufficient quantitative detail, because some currently return only tumour fraction estimates, composite scores, or binary MRD classifications. Third, broader uptake would depend on agreement across clinicians, laboratories, and clinical trial groups regarding minimum reporting elements, interpretive categories, and candidate thresholds for prospective validation. A practical first step would be to require reporting of KRAS mutant copies or molecules per mL as a mandatory field in prospective clinical trial protocols, while encouraging professional societies, regulatory bodies, and reporting consortia to consider this metric within minimum ctDNA reporting standards. It simply makes both absolute burden and measurement uncertainty explicit in cases where the biology permits. For clarity, we refer to aggregate ctDNA measurements interpreted relative to a prior KRAS anchor as being expressed in *KRAS-equivalent molecules per millilitre*, recognising that these represent inferred, calibration-dependent quantities rather than direct measurements of KRAS below the limit of quantification.

**Figure 2:**
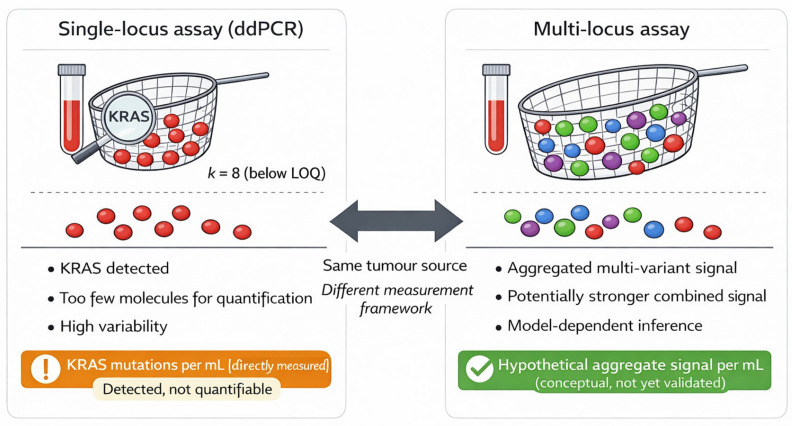
Conceptual schematic contrasting direct single-locus quantification with inferred multi-locus aggregation. The left panel illustrates direct measurement of mutant KRAS molecules in a single-locus assay, where very low molecule counts may permit detection but remain below the limit of quantification (LOQ). The right panel illustrates a hypothetical multi-locus framework in which signals from multiple tumour-derived variants are aggregated to strengthen the overall ctDNA signal. This panel is shown as a conceptual illustration only, intended to distinguish direct molecule counting from model-based aggregate inference. It does not imply that a calibrated “KRAS-equivalent” or other volume-normalised aggregate metric has been established or standardised across PDAC assays or platforms. Thresholds and labels are illustrative only.

Figure 2 illustrates how identical underlying tumour biology can yield different quantitative confidence depending on assay strategy. In single-locus KRAS assay, low absolute molecule counts may result in a detectable but non-quantifiable measurement despite direct measurement of KRAS mutant molecules per millilitre. In contrast, multi-locus assays aggregate signal across multiple tumour-derived variants, allowing quantifiable estimates to be derived even when KRAS itself falls below the limit of quantification provided sufficient total mutant molecules are captured.

### 4.5. Implications for Interoperability and Assay Choice

Anchoring aggregate ctDNA metrics to KRAS when possible improves interpretability across platforms and studies. It facilitates comparison between assays that may otherwise appear discordant and supports cumulative learning from heterogeneous data sources. However, cross-platform comparability cannot be assumed without analytical validation. Differences in molecular capture efficiency, library preparation, sequencing depth, background error rates, and variant-calling rules may all influence the quantitative accuracy of KRAS-derived estimates across assays. For this reason, calibration should ideally be evaluated using shared reference materials, contrived control samples, or split-sample comparisons across platforms to determine whether KRAS molecules/mL or KRAS-equivalent molecules/mL remain stable under different technical conditions. Such studies would help define when biological interoperability is sufficient for clinical interpretation and when platform-specific correction or reporting constraints are still required.

Critically, interoperability need not be assumed a priori. Repeated calibration to quantifiable KRAS across patients, timepoints, and platforms would allow empirical confirmation that diverse ctDNA assays are sampling the same underlying biological signal. As such calibration accumulates, confidence that different aggregate metrics are estimating a shared quantity increases, enabling principled cross-study synthesis and data pooling rather than parallel, non-comparable reporting.

This framework also allows the rational coexistence of different assay strategies. Broad multi-locus sequencing may be required for low-shedding disease or very low ctDNA levels, while targeted approaches such as KRAS-focused assays may be sufficient—and more economical—for serial monitoring in higher-shedding patients. Expressing results on a shared biological reference scale enables such choices without fragmenting interpretation. In metastatic disease monitoring, this would also improve continuity of interpretation when patients change centres, countries, or providers, because serial ctDNA results generated by different assays could be mapped onto a common KRAS-anchored scale rather than being interpreted as isolated platform-specific values.

### 4.6. Clinical Implications

A KRAS-based absolute reporting framework could influence clinical development and reporting in several concrete ways. First, in postoperative MRD studies, results could be prospectively stratified as not detected, detectable but not quantifiable, and quantifiable, rather than collapsing all low-level signals into a binary positive/negative category. This would allow adjuvant trial designs to test whether low-level detectable KRAS below the limit of quantification identifies an intermediate-risk group with residual disease concern. Second, in metastatic disease monitoring, reporting KRAS mutant molecules per millilitre could improve cross-platform continuity by allowing serial ctDNA results from different assays or centres to be interpreted on a common biological scale. More broadly, this approach could support future guideline-level recommendations for minimum reporting standards, including mutant molecule count, plasma-equivalent input volume, derived concentration, and explicit distinction between detectability and quantifiability.

### 4.7. Broader Relevance

Most ctDNA platforms are designed to operate across multiple tumour types, and solutions that rely on a near-ubiquitous truncal alteration would not naturally arise from a pan-oncology development perspective. The approach outlined here therefore reflects a disease-specific opportunity rather than a universal solution.

Viewed more abstractly, KRAS-equivalent mutations per millilitre in pancreatic cancer may be understood as a specific instantiation of a more general quantitative objective: expressing aggregate ctDNA signal in units that approximate an underlying burden of tumour-derived DNA entering the circulation. This proposal is aligned with, rather than being orthogonal to, existing liquid biopsy guidance that emphasises analytical transparency, clinical interpretability, and fit-for-purpose reporting. In other tumour types such as EGFR-mutant NSCLC, absolute mutant copies per millilitre have already been used in some assay and monitoring contexts, supporting the broader principle that molecule-based outputs can aid longitudinal interpretation. In PDAC, no widely adopted ctDNA guideline currently standardises reporting around KRAS copies/mL, and current practice remains dominated by binary detection, VAF-like measures, or proprietary composite outputs. The present framework therefore extends the direction of existing guidance by proposing a PDAC-specific biological anchor that could help harmonise reporting rather than challenge established clinical principles. Pancreatic cancer provides an unusually tractable setting in which this objective can be examined empirically, owing to the availability of a near-universal truncal reference. Demonstrating convergence on KRAS-equivalent units in this context would support the interpretation that diverse ctDNA algorithms are estimating a shared underlying quantity, with implications for how analogous anchor-equivalent frameworks might be constructed in other tumour types.

Thus, pancreatic cancer may serve as a practical testbed for examining how aggregate ctDNA metrics relate to truncal biological signals. Demonstrating consistency in this setting could increase confidence in the interpretability of quantitative ctDNA outputs more broadly, even where analogous anchors are unavailable.

## 5. Conclusions

This evidence-mapping review demonstrates that quantitative ctDNA reporting in PDAC remains heterogeneous and lacks a shared biological reference framework. Across single-locus *KRAS* assays, multi-locus sequencing platforms, and published meta-analyses, tumour burden is variably expressed as variant allele frequency, tumour fraction, proprietary aggregate scores, or binary detectability, with limited standardisation of plasma input volume, absolute molecule counts, or limits of quantification. As a result, quantitative ctDNA data in PDAC accumulate in parallel rather than cumulatively, constraining cross-study synthesis and impeding metrological clarity.

Pancreatic cancer offers a unique opportunity to address this limitation. The near-universal presence of truncal activating *KRAS* mutations provides a biologically grounded calibration axis that is already embedded (explicitly or implicitly) within most ctDNA assays applied to this disease. Reporting *KRAS* mutant molecules per millilitre, together with explicit plasma-equivalent input volume and acknowledgment of quantitative uncertainty, enables ctDNA measurements to be interpreted on a statistically coherent and biologically meaningful scale.

We propose a minimal, assay-agnostic framework that distinguishes detectability from quantifiability based on counting statistics, while allowing aggregate multi-locus outputs to be expressed in KRAS-equivalent units when calibration is feasible. This approach does not mandate changes to analytical pipelines, nor does it privilege a specific platform. Rather, it makes explicit the biological coordinate system in which ctDNA measurements are already being interpreted. If adopted, this framework could improve interoperability, enable principled cross-platform comparison, and support cumulative learning in pancreatic cancer ctDNA research. More broadly, PDAC may serve as a practical testbed for evaluating how aggregate ctDNA metrics relate to truncal biological anchors, informing the development of more interpretable quantitative standards in liquid biopsy oncology. The review therefore supports direct count-based interpretability most strongly in single-locus settings, whereas the extension of similar quantitative language to broader assay classes should presently be viewed as hypothesis-generating rather than standard-setting.

## Figures and Tables

**Table 2:** Characteristics of published meta-analyses evaluating ctDNA in PDAC. Published meta-analyses pooled binary or study-specific relative ctDNA measures, and none proposed a harmonised absolute quantitative framework across assays. Entries listed as “not reported” indicate absence of reporting in the original publication after full-text review, rather than omission during data extraction.

Meta-Analysis	Main Outcomes Pooled	What was Actually Pooled?	Any Quantitative Calibration Across Studies/Platforms?	Any Absolute ctDNA Threshold Proposed?
Steiniche et al. [57]	Prognosis in non-resectable PDAC (OS/PFS), ctDNA kinetics	HRs for “high baseline ctDNA” and “unfavourable kinetics”; acknowledges study-specific thresholds	No	No
Zheng et al. [58]	Early diagnosis (stage I–II focus)	Bivariate diagnostic meta-analysis (sens/spec/DOR/AUC), includes ctDNA subgroup	No	No
Alqahtani et al. [59]	Prognosis in resected PDAC (OS/RFS)	HRs for KRAS-mutated ctDNA-positive vs. negative (pre-/post-op)	No	No
Vidal et al. [60]	Liquid biopsy around surgery (OS/DFS)	HRs based on ctDNA status shifts (−/+; +/−)	No	No
Bunduc et al. [61]	Prognosis: cfDNA/ctDNA markers (OS/PFS)	HRs for detectable ctDNA and KRAS mutation detection	No	No
Guven et al. [62]	Prognosis (localised + advanced)	HRs for ctDNA-positive vs. negative at different timepoints	No	No
Milin-Lazovic et al. [63]	Prognosis: cfDNA/ctDNA and KRAS	HRs for cfDNA/ctDNA positivity and KRAS presence	No	No
Fang et al. [64]	Prognosis (OS/PFS)	HRs for “mutations detected” and/or “high concentration” (not harmonised)	No	No
Zhu et al. [65]	Diagnostic value of liquid biopsy (ctDNA/CTCs/exosomes)	Pooled sensitivity/specificity/AUC for ctDNA subgroup	No	No
Lee et al. [66]	Prognosis in resectable PDAC	HRs for ctDNA detectable vs. not (baseline/post-op)	No	No
Chen et al. [67]	Prognosis in pancreatic cancer: OS, PFS, and DSS	Hazard ratios for OS/PFS/DSS according to cfDNA-related variables, including mutation status, ctDNA presence, hypermethylation, and higher cfDNA concentration	No	No
Borges et al. [68]	Prognosis in resected PDAC: DFS and OS in preoperative and postoperative settings; subgroup analysis by upfront surgery and neoadjuvant treatment	Hazard ratios comparing preoperative or postoperative ctDNA-positive versus ctDNA-negative patients for DFS and OS; subgroup analysis by upfront surgery and neoadjuvant treatment	No	No

Abbreviations: AUC, area under the curve; cfDNA, cell-free DNA; CTCs, circulating tumour cells; ctDNA, circulating tumour DNA; DFS, disease-free survival; DOR, diagnostic odds ratio; HRs, hazard ratios; KRAS, Kirsten rat sarcoma viral oncogene homologue; OS, overall survival; PDAC, pancreatic ductal adenocarcinoma; PFS, progression-free survival; RFS, recurrence-free survival.

## Data Availability

All data generated in this study were presented in the manuscript.

## Supplementary Materials

The following supporting information can be downloaded at: https://www.mdpi.com/article/10.3390/cancers18081318/s1, File S1: PRISMA 2020 checklist for the study titled: Toward a Shared Quantitative Language for ctDNA in Pancreatic Cancer [80]. File S2: Full electronic search strategies for the study titled “Toward a Shared Quantitative Language for ctDNA in Pancreatic Cancer”.

## Abbreviations

The following abbreviations are used in this manuscript:

cfDNA	Cell-free DNA
ctDNA	Circulating tumour DNA
CV	Coefficient of variation
ddPCR	Droplet digital polymerase chain reaction
LoD	Limit of detection
LoQ	Limit of quantification
MRD	Minimal residual disease
NGS	Next-generation sequencing
PDAC	Pancreatic ductal adenocarcinoma
PRISMA	Preferred Reporting Items for Systematic Reviews and Meta-Analyses
VAF	Variant allele frequency
cfDNA	Cell-free DNA

## References

[1] Diehl F., Schmidt K., Choti M.A., Romans K., Goodman S., Li M., Thornton K., Agrawal N., Sokoll L., Szabo S.A. (2008). Circulating mutant DNA to assess tumor dynamics. Nat. Med..

[2] Dawson S.-J., Tsui D.W., Murtaza M., Biggs H., Rueda O.M., Chin S.-F., Dunning M.J., Gale D., Forshew T., Mahler-Araujo B. (2013). Analysis of circulating tumor DNA to monitor metastatic breast cancer. N. Engl. J. Med..

[3] Bettegowda C., Sausen M., Leary R.J., Kinde I., Wang Y., Agrawal N., Bartlett B.R., Wang H., Luber B., Alani R.M. (2014). Detection of circulating tumor DNA in early-and late-stage human malignancies. Sci. Transl. Med..

[4] Tie J., Wang Y., Tomasetti C., Li L., Springer S., Kinde I., Silliman N., Tacey M., Wong H.-L., Christie M. (2016). Circulating tumor DNA analysis detects minimal residual disease and predicts recurrence in patients with stage II colon cancer. Sci. Transl. Med..

[5] Murtaza M., Dawson S.-J., Tsui D.W., Gale D., Forshew T., Piskorz A.M., Parkinson C., Chin S.-F., Kingsbury Z., Wong A.S. (2013). Non-invasive analysis of acquired resistance to cancer therapy by sequencing of plasma DNA. Nature.

[6] Merker J.D., Oxnard G.R., Compton C., Diehn M., Hurley P., Lazar A.J., Lindeman N., Lockwood C.M., Rai A.J., Schilsky R.L. (2018). Circulating tumor DNA analysis in patients with cancer: American Society of Clinical Oncology and College of American Pathologists joint review. Arch. Pathol. Lab. Med..

[7] Terrault N.A., Bzowej N.H., Chang K.M., Hwang J.P., Jonas M.M., Murad M.H. (2016). A ASLD guidelines for treatment of chronic hepatitis B. Hepatology.

[8] Lampertico P., Agarwal K., Berg T., Buti M., Janssen H.L., Papatheodoridis G., Zoulim F., Tacke F. (2017). EASL 2017 Clinical Practice Guidelines on the management of hepatitis B virus infection. J. Hepatol..

[9] Baylis S., Wallace P., McCulloch E., Niesters H., Nübling C. (2019). Standardization of nucleic acid tests: The approach of the World Health Organization. J. Clin. Microbiol..

[10] Wong G.L.-H., Lemoine M. (2025). The 2024 updated WHO guidelines for the prevention and management of chronic hepatitis B: Main changes and potential implications for the next major liver society clinical practice guidelines. J. Hepatol..

[11] Lai C.-L., Yuen M.-F. (2007). The natural history and treatment of chronic hepatitis B: A critical evaluation of standard treatment criteria and end points. Ann. Intern. Med..

[12] Wan J.C., Massie C., Garcia-Corbacho J., Mouliere F., Brenton J.D., Caldas C., Pacey S., Baird R., Rosenfeld N. (2017). Liquid biopsies come of age: Towards implementation of circulating tumour DNA. Nat. Rev. Cancer.

[13] Heitzer E., Ulz P., Geigl J.B. (2015). Circulating tumor DNA as a liquid biopsy for cancer. Clin. Chem..

[14] Corcoran R.B., Chabner B.A. (2018). Application of cell-free DNA analysis to cancer treatment. N. Engl. J. Med..

[15] Vogelstein B., Kinzler K.W. (1999). Digital pcr. Proc. Natl. Acad. Sci. USA.

[16] Hindson B.J., Ness K.D., Masquelier D.A., Belgrader P., Heredia N.J., Makarewicz A.J., Bright I.J., Lucero M.Y., Hiddessen A.L., Legler T.C. (2011). High-throughput droplet digital PCR system for absolute quantitation of DNA copy number. Anal. Chem..

[17] Newman A.M., Bratman S.V., To J., Wynne J.F., Eclov N.C., Modlin L.A., Liu C.L., Neal J.W., Wakelee H.A., Merritt R.E. (2014). An ultrasensitive method for quantitating circulating tumor DNA with broad patient coverage. Nat. Med..

[18] Phallen J., Sausen M., Adleff V., Leal A., Hruban C., White J., Anagnostou V., Fiksel J., Cristiano S., Papp E. (2017). Direct detection of early-stage cancers using circulating tumor DNA. Sci. Transl. Med..

[19] Razavi P., Li B.T., Brown D.N., Jung B., Hubbell E., Shen R., Abida W., Juluru K., De Bruijn I., Hou C. (2019). High-intensity sequencing reveals the sources of plasma circulating cell-free DNA variants. Nat. Med..

[20] Cohen J.D., Javed A.A., Thoburn C., Wong F., Tie J., Gibbs P., Schmidt C.M., Yip-Schneider M.T., Allen P.J., Schattner M. (2017). Combined circulating tumor DNA and protein biomarker-based liquid biopsy for the earlier detection of pancreatic cancers. Proc. Natl. Acad. Sci. USA.

[21] Brychta N., Krahn T., von Ahsen O. (2016). Detection of KRAS mutations in circulating tumor DNA by digital PCR in early stages of pancreatic cancer. Clin. Chem..

[22] Chen I., Raymond V.M., Geis J.A., Collisson E.A., Jensen B.V., Hermann K.L., Erlander M.G., Tempero M., Johansen J.S. (2017). Ultrasensitive plasma ctDNA KRAS assay for detection, prognosis, and assessment of therapeutic response in patients with unresectable pancreatic ductal adenocarcinoma. Oncotarget.

[23] Guo S., Shi X., Shen J., Gao S., Wang H., Shen S., Pan Y., Li B., Xu X., Shao Z. (2020). Preoperative detection of KRAS G12D mutation in ctDNA is a powerful predictor for early recurrence of resectable PDAC patients. Br. J. Cancer.

[24] Macgregor-Das A., Yu J., Tamura K., Abe T., Suenaga M., Shindo K., Borges M., Koi C., Kohi S., Sadakari Y. (2020). Detection of circulating tumor DNA in patients with pancreatic cancer using digital next-generation sequencing. J. Mol. Diagn..

[25] van der Sijde F., Azmani Z., Besselink M.G., Bonsing B.A., de Groot J.W.B., Groot Koerkamp B., Haberkorn B.C., Homs M.Y., van IJcken W.F., Janssen Q.P. (2021). Circulating TP53 mutations are associated with early tumor progression and poor survival in pancreatic cancer patients treated with FOLFIRINOX. Ther. Adv. Med. Oncol..

[26] Wang R., Zhao Y., Wang Y., Zhao Z., Chen Q., Duan Y., Xiong S., Luan Z., Wang J., Cheng B. (2022). Diagnostic and prognostic values of KRAS mutations on EUS-FNA specimens and circulating tumor DNA in patients with pancreatic cancer. Clin. Transl. Gastroenterol..

[27] Watanabe K., Nakamura T., Kimura Y., Motoya M., Kojima S., Kuraya T., Murakami T., Kaneko T., Shinohara Y., Kitayama Y. (2022). Tumor-informed approach improved ctDNA detection rate in resected pancreatic cancer. Int. J. Mol. Sci..

[28] Hata T., Mizuma M., Motoi F., Ohtsuka H., Nakagawa K., Morikawa T., Unno M. (2023). Prognostic impact of postoperative circulating tumor DNA as a molecular minimal residual disease marker in patients with pancreatic cancer undergoing surgical resection. J. Hepato-Biliary-Pancreat. Sci..

[29] Levink I.J., Jansen M.P., Azmani Z., van IJcken W., van Marion R., Peppelenbosch M.P., Cahen D.L., Fuhler G.M., Bruno M.J. (2023). Mutation analysis of pancreatic juice and plasma for the detection of pancreatic cancer. Int. J. Mol. Sci..

[30] Lee M.R., Woo S.M., Kim M.K., Han S.S., Park S.J., Lee W.J., Lee D.e., Choi S.I., Choi W., Yoon K.A. (2024). Application of plasma circulating KRAS mutations as a predictive biomarker for targeted treatment of pancreatic cancer. Cancer Sci..

[31] Murakami T., Imamura M., Kimura Y., Watanabe K., Shinohara Y., Nakamura T., Low S.-K., Motoya M., Kawakami Y., Masaki Y. (2025). Role of preoperative circulating tumor DNA in predicting occult metastases in resectable and borderline resectable pancreatic ductal adenocarcinoma. World J. Gastroenterol..

[32] Zhang Y., Esmail A., Hassanain H., Dhillon V., Abdelrahim W., Al-Najjar E., Khasawneh B., Abdelrahim M. (2025). Correlative analysis of tumor-Informed Circulating tumor DNA (ctDNA) and the survival outcomes of patients with pancreatic adenocarcinoma. Biomedicines.

[33] Kinugasa H., Nouso K., Miyahara K., Morimoto Y., Dohi C., Tsutsumi K., Kato H., Matsubara T., Okada H., Yamamoto K. (2015). Detection of K-ras gene mutation by liquid biopsy in patients with pancreatic cancer. Cancer.

[34] Takai E., Totoki Y., Nakamura H., Morizane C., Nara S., Hama N., Suzuki M., Furukawa E., Kato M., Hayashi H. (2015). Clinical utility of circulating tumor DNA for molecular assessment in pancreatic cancer. Sci. Rep..

[35] Tjensvoll K., Lapin M., Buhl T., Oltedal S., Berry K.S.-O., Gilje B., Søreide J.A., Javle M., Nordgård O., Smaaland R. (2016). Clinical relevance of circulating KRAS mutated DNA in plasma from patients with advanced pancreatic cancer. Mol. Oncol..

[36] Berger A.W., Schwerdel D., Ettrich T.J., Hann A., Schmidt S.A., Kleger A., Marienfeld R., Seufferlein T. (2017). Targeted deep sequencing of circulating tumor DNA in metastatic pancreatic cancer. Oncotarget.

[37] Pietrasz D., Pecuchet N., Garlan F., Didelot A., Dubreuil O., Doat S., Imbert-Bismut F., Karoui M., Vaillant J.-C., Taly V. (2017). Plasma circulating tumor DNA in pancreatic cancer patients is a prognostic marker. Clin. Cancer Res..

[38] Kruger S., Heinemann V., Ross C., Diehl F., Nagel D., Ormanns S., Liebmann S., Prinz-Bravin I., Westphalen C., Haas M. (2018). Repeated mutKRAS ctDNA measurements represent a novel and promising tool for early response prediction and therapy monitoring in advanced pancreatic cancer. Ann. Oncol..

[39] Perets R., Greenberg O., Shentzer T., Semenisty V., Epelbaum R., Bick T., Sarji S., Ben-Izhak O., Sabo E., Hershkovitz D. (2018). Mutant KRAS circulating tumor DNA is an accurate tool for pancreatic cancer monitoring. Oncologist.

[40] Wang Z.-Y., Ding X.-Q., Zhu H., Wang R.-X., Pan X.-R., Tong J.-H. (2019). KRAS mutant allele fraction in circulating cell-free DNA correlates with clinical stage in pancreatic cancer patients. Front. Oncol..

[41] Wei T., Zhang Q., Li X., Su W., Li G., Ma T., Gao S., Lou J., Que R., Zheng L. (2019). Monitoring tumor burden in response to FOLFIRINOX chemotherapy via profiling circulating cell-free DNA in pancreatic cancer. Mol. Cancer Ther..

[42] Strijker M., Soer E.C., de Pastena M., Creemers A., Balduzzi A., Beagan J.J., Busch O.R., van Delden O.M., Halfwerk H., van Hooft J.E. (2020). Circulating tumor DNA quantity is related to tumor volume and both predict survival in metastatic pancreatic ductal adenocarcinoma. Int. J. Cancer.

[43] Wei T., Zhang J., Li J., Chen Q., Zhi X., Tao W., Ma J., Yang J., Lou Y., Ma T. (2020). Genome-wide profiling of circulating tumor DNA depicts landscape of copy number alterations in pancreatic cancer with liver metastasis. Mol. Oncol..

[44] Takano S., Fukasawa M., Shindo H., Takahashi E., Fukasawa Y., Kawakami S., Hayakawa H., Kuratomi N., Kadokura M., Maekawa S. (2021). Digital next-generation sequencing of cell-free DNA for pancreatic cancer. JGH Open.

[45] Botrus G., Uson Junior P.L.S., Raman P., Kaufman A.E., Kosiorek H., Yin J., Fu Y., Majeed U., Sonbol M.B., Ahn D.H. (2022). Circulating cell-free tumor DNA in advanced pancreatic adenocarcinoma identifies patients with worse overall survival. Front. Oncol..

[46] Lee J.-S., Han Y., Yun W.-G., Kwon W., Kim H., Jeong H., Seo M.-S., Park Y., Cho S.I., Kim H. (2022). Parallel analysis of pre-and postoperative circulating tumor DNA and matched tumor tissues in resectable pancreatic ductal adenocarcinoma: A prospective cohort study. Clin. Chem..

[47] Lapin M., Edland K.H., Tjensvoll K., Oltedal S., Austdal M., Garresori H., Rozenholc Y., Gilje B., Nordgård O. (2023). Comprehensive ctDNA measurements improve prediction of clinical outcomes and enable dynamic tracking of disease progression in advanced pancreatic cancer. Clin. Cancer Res..

[48] Tanaka J., Nakagawa T., Harada K., Morizane C., Tanaka H., Shiba S., Ohba A., Hijioka S., Takai E., Yachida S. (2023). Efficient and accurate KRAS genotyping using digital PCR combined with melting curve analysis for ctDNA from pancreatic cancer patients. Sci. Rep..

[49] Huerta M., Martín-Arana J., Gimeno-Valiente F., Carbonell-Asins J.A., García-Micó B., Martínez-Castedo B., Robledo-Yagüe F., Camblor D.G., Fleitas T., Bartolomé M.G. (2024). ctDNA whole exome sequencing in pancreatic ductal adenocarcinoma unveils organ-dependent metastatic mechanisms and identifies actionable alterations in fast progressing patients. Transl. Res..

[50] Theparee T., Akroush M., Sabatini L.M., Wang V., Mangold K.A., Joseph N., Stocker S.J., Freedman A., Helseth D.L., Talamonti M.S. (2024). Cell free DNA in patients with pancreatic adenocarcinoma: Clinicopathologic correlations. Sci. Rep..

[51] Petersson A., Svensson M., Hau S.O., Bergström R., Lindberg J., Mayrhofer M., Chattopadhyay S., Eberhard J., Heidenblad M., Leandersson K. (2025). Reliable on-treatment prognostication and target identification with a customized assay for circulating tumor DNA in patients with newly diagnosed pancreatic cancer. Sci. Rep..

[52] Zavrtanik Čarni H., Badovinac D., Blagus T., Goričar K., Ranković B., Matjašič A., Zupan A., Tomažič A., Dolžan V. (2025). Somatic mutation detection in tumor tissue and matched Cell-Free DNA using PCR-Based methods in pancreatic cancer patients undergoing upfront resection. Int. J. Mol. Sci..

[53] Hadano N., Murakami Y., Uemura K., Hashimoto Y., Kondo N., Nakagawa N., Sueda T., Hiyama E. (2016). Prognostic value of circulating tumour DNA in patients undergoing curative resection for pancreatic cancer. Br. J. Cancer.

[54] Lin M., Alnaggar M., Liang S., Chen J., Xu K., Dong S., Du D., Niu L. (2018). Circulating tumor DNA as a sensitive marker in patients undergoing irreversible electroporation for pancreatic cancer. Cell. Physiol. Biochem..

[55] Hussung S., Hess M.E., Haghighi E.B., Wittel U.A., Boerries M., Fritsch R.M. (2024). Integrated analysis of cell-free DNA and novel protein biomarkers for stratification and therapy monitoring in stage IV pancreatic cancer: A preliminary study. Diagnostics.

[56] Sellahewa R., Moghaddam S.M., Lundy J., Jenkins B.J., Croagh D. (2023). Circulating tumor DNA is an accurate diagnostic tool and strong prognostic marker in pancreatic cancer. Pancreas.

[57] Steiniche M.M., Callesen L.B., Vlk E.H., Ventzel L., Timm S., Andersen R.F., Lindgaard S.C., Hansen T.F., Ladekarl M., Spindler K.-L.G. (2025). Circulating tumour DNA (ctDNA) as a predictor of progression-free and overall survival in non-resectable pancreatic cancer: A systematic review and meta-analysis. J. Liq. Biopsy.

[58] Zheng Z., Lu Z., Yan F., Song Y. (2025). The role of novel biomarkers in the early diagnosis of pancreatic cancer: A systematic review and meta-analysis. PLoS ONE.

[59] Alqahtani A., Alloghbi A., Coffin P., Yin C., Mukherji R., Weinberg B.A. (2023). Prognostic utility of preoperative and postoperative KRAS-mutated circulating tumor DNA (ctDNA) in resected pancreatic ductal adenocarcinoma: A systematic review and meta-analysis. Surg. Oncol..

[60] Vidal L., Pando E., Blanco L., Fabregat-Franco C., Castet F., Sierra A., Macarulla T., Balsells J., Charco R., Vivancos A. (2023). Liquid biopsy after resection of pancreatic adenocarcinoma and its relation to oncological outcomes. Systematic review and meta-analysis. Cancer Treat. Rev..

[61] Bunduc S., Gede N., Vancsa S., Lillik V., Kiss S., Dembrovszky F., Eross B., Szakacs Z., Gheorghe C., Miko A. (2022). Prognostic role of cell-free DNA biomarkers in pancreatic adenocarcinoma: A systematic review and meta–analysis. Crit. Rev. Oncol./Hematol..

[62] Guven D.C., Sahin T.K., Yildirim H.C., Aktepe O.H., Dizdar O., Yalcin S. (2021). A systematic review and meta-analysis of the association between circulating tumor DNA (ctDNA) and prognosis in pancreatic cancer. Crit. Rev. Oncol./Hematol..

[63] Milin-Lazovic J., Madzarevic P., Rajovic N., Djordjevic V., Milic N., Pavlovic S., Veljkovic N., Milic N.M., Radenkovic D. (2021). Meta-analysis of circulating cell-free DNA’s role in the prognosis of pancreatic cancer. Cancers.

[64] Fang Z., Meng Q., Zhang B., Shi S., Liu J., Liang C., Hua J., Yu X., Xu J., Wang W. (2020). Prognostic value of circulating tumor DNA in pancreatic cancer: A systematic review and meta-analysis. Aging.

[65] Zhu Y., Zhang H., Chen N., Hao J., Jin H., Ma X. (2020). Diagnostic value of various liquid biopsy methods for pancreatic cancer: A systematic review and meta-analysis. Medicine.

[66] Lee J.-S., Rhee T.-M., Pietrasz D., Bachet J.-B., Laurent-Puig P., Kong S.-Y., Takai E., Yachida S., Shibata T., Lee J.W. (2019). Circulating tumor DNA as a prognostic indicator in resectable pancreatic ductal adenocarcinoma: A systematic review and meta-analysis. Sci. Rep..

[67] Borges F.C., Pinto M.S., Borges M.F., João A.A., Francisco E., Sousa M., Aral M., Oliveira V., Cunha J.F., Mehrabi A. (2026). The Role of Circulating Tumor DNA in Surgical Management of Pancreatic Cancer: Systematic Review and Meta-analysis. Ann. Surg..

[68] Chen L., Zhang Y., Cheng Y., Zhang D., Zhu S., Ma X. (2018). Prognostic value of circulating cell-free DNA in patients with pancreatic cancer: A systemic review and meta-analysis. Gene.

[69] Rolfo C., Mack P., Scagliotti G.V., Aggarwal C., Arcila M.E., Barlesi F., Bivona T., Diehn M., Dive C., Dziadziuszko R. (2021). Liquid biopsy for advanced NSCLC: A consensus statement from the international association for the study of lung cancer. J. Thorac. Oncol..

[70] Esagian S.M., Grigoriadou G.Ι., Nikas I.P., Boikou V., Sadow P.M., Won J.-K., Economopoulos K.P. (2020). Comparison of liquid-based to tissue-based biopsy analysis by targeted next generation sequencing in advanced non-small cell lung cancer: A comprehensive systematic review. J. Cancer Res. Clin. Oncol..

[71] Siravegna G., Marsoni S., Siena S., Bardelli A. (2017). Integrating liquid biopsies into the management of cancer. Nat. Rev. Clin. Oncol..

[72] Zill O.A., Greene C., Sebisanovic D., Siew L.M., Leng J., Vu M., Hendifar A.E., Wang Z., Atreya C.E., Kelley R.K. (2015). Cell-free DNA next-generation sequencing in pancreatobiliary carcinomas. Cancer Discov..

[73] Reinert T., Petersen L.M.S., Henriksen T.V., Larsen M.Ø., Rasmussen M.H., Johansen A.F.B., Øgaard N., Knudsen M., Nordentoft I., Vang S. (2022). Circulating tumor DNA for prognosis assessment and postoperative management after curative-intent resection of colorectal liver metastases. Int. J. Cancer.

[74] McDonald B.R., Contente-Cuomo T., Sammut S.J., Odenheimer-Bergman A., Ernst B., Perdigones N., Chin S.F., Farooq M., Mejia R., Cronin P.A. (2019). Personalized circulating tumor DNA analysis to detect residual disease after neoadjuvant therapy in breast cancer. Sci. Transl. Med..

[75] Henriksen T.V.V., Tarazona N., Frydendahl A., Reinert T., Carbonell-Asins J.A., Sharma S., Renner D., Roda D., Huerta M., Rosello S. (2021). Serial circulating tumor DNA analysis to assess recurrence risk, benefit of adjuvant therapy, growth rate and early relapse detection in stage III colorectal cancer patients. J. Clin. Oncol..

[76] Raphael B.J., Hruban R.H., Aguirre A.J., Moffitt R.A., Yeh J.J., Stewart C., Robertson A.G., Cherniack A.D., Gupta M., Getz G. (2017). Integrated genomic characterization of pancreatic ductal adenocarcinoma. Cancer Cell.

[77] Biankin A.V., Waddell N., Kassahn K.S., Gingras M.-C., Muthuswamy L.B., Johns A.L., Miller D.K., Wilson P.J., Patch A.-M., Wu J. (2012). Pancreatic cancer genomes reveal aberrations in axon guidance pathway genes. Nature.

[78] Waddell N., Pajic M., Patch A.-M., Chang D.K., Kassahn K.S., Bailey P., Johns A.L., Miller D., Nones K., Quek K. (2015). Whole genomes redefine the mutational landscape of pancreatic cancer. Nature.

[79] Bailey P., Chang D.K., Nones K., Johns A.L., Patch A.-M., Gingras M.-C., Miller D.K., Christ A.N., Bruxner T.J., Quinn M.C. (2016). Genomic analyses identify molecular subtypes of pancreatic cancer. Nature.

[80] Page M.J., McKenzie J.E., Bossuyt P.M., Boutron I., Hoffmann T.C., Mulrow C.D., Shamseer L., Tetzlaff J.M., Akl E.A., Brennan S.E. (2021). The PRISMA 2020 statement: An updated guideline for reporting systematic reviews. BMJ.

