# Supplementary material for: Evidence Mapping of ctDNA Reporting in Pancreatic Ductal Adenocarcinoma: Toward a Shared Quantitative Language for ctDNA
Source: Cancers (Basel). 2026 Apr 21;18(8):1318. doi: 10.3390/cancers18081318 (PMC13115254; doi:10.3390/cancers18081318)
Supplement: Supplementary file 1 [file cancers-18-01318-s001.zip › Supplementary File S2_search_strategies 10042026-SA 12042026 DC latest.pdf]

File S2. Full electronic search strategies for the study titled “Toward a Shared Quantitative Language for ctDNA in Pancreatic Cancer”.

PubMed/MEDLINE

Field	Content	Records
**A. KRAS ddPCR-focused primary studies**	(("pancreatic ductal adenocarcinoma"[Title/Abstract] OR "pancreatic cancer"[Title/Abstract] OR PDAC[Title/Abstract])) AND (KRAS[Title/Abstract]) AND ("circulating tumor DNA"[Title/Abstract] OR "circulating tumour DNA"[Title/Abstract] OR ctDNA[Title/Abstract]) AND ("digital PCR"[Title/Abstract] OR "droplet digital PCR"[Title/Abstract] OR ddPCR[Title/Abstract] OR BEAMing[Title/Abstract]) AND (plasma[Title/Abstract]) NOT (review[Publication Type] OR meta-analysis[Publication Type])	18
**B. NGS/panel-based primary studies**	(("pancreatic ductal adenocarcinoma"[Title/Abstract] OR "pancreatic cancer"[Title/Abstract] OR PDAC[Title/Abstract])) AND ("circulating tumor DNA"[Title/Abstract] OR "circulating tumour DNA"[Title/Abstract] OR ctDNA[Title/Abstract] OR "liquid biopsy"[Title/Abstract]) AND ("next generation sequencing"[Title/Abstract] OR NGS[Title/Abstract] OR "gene panel"[Title/Abstract] OR "tumor fraction"[Title/Abstract] OR "tumour fraction"[Title/Abstract]) AND (plasma[Title/Abstract]) NOT (KRAS[Title/Abstract] AND ddPCR[Title/Abstract]) NOT (review[Publication Type] OR meta-analysis[Publication Type])	28
**C. Systematic reviews and meta-analyses**	(("pancreatic ductal adenocarcinoma"[Title/Abstract] OR "pancreatic cancer"[Title/Abstract] OR PDAC[Title/Abstract])) AND ("circulating tumor DNA"[Title/Abstract] OR "circulating tumour DNA"[Title/Abstract] OR ctDNA[Title/Abstract]) AND (meta-analysis[Title/Abstract] OR "systematic review and meta-analysis"[Title/Abstract])	14

Scopus

Field	Content	Records
**A. KRAS ddPCR-focused primary studies**	TITLE-ABS(("pancreatic ductal adenocarcinoma" OR "pancreatic cancer" OR PDAC) AND KRAS AND ("circulating tumor DNA" OR "circulating tumour DNA" OR ctDNA) AND ("digital PCR" OR "droplet digital PCR" OR ddPCR OR BEAMing) AND plasma) AND NOT TITLE-ABS(review OR "meta-analysis") AND NOT DOCTYPE(re)	18
**B. NGS/panel-based primary studies**	TITLE-ABS(("pancreatic ductal adenocarcinoma" OR "pancreatic cancer" OR PDAC) AND ("circulating tumor DNA" OR "circulating tumour DNA" OR ctDNA OR "liquid biopsy") AND ("next generation sequencing" OR NGS OR "gene panel" OR "tumor fraction" OR "tumour fraction") AND plasma) AND NOT TITLE-ABS(KRAS AND ddPCR) AND NOT TITLE-ABS(review OR "meta-analysis") AND NOT DOCTYPE(re)	27

**C. Systematic reviews and meta-analyses**	TITLE-ABS(("pancreatic ductal adenocarcinoma" OR "pancreatic cancer" OR PDAC) AND ("circulating tumor DNA" OR "circulating tumour DNA" OR ctDNA) AND ("meta-analysis" OR "systematic review and meta-analysis"))	17

A. Shared duplicate titles across PubMed and Scopus (n = 42)

1. A Multianalyte Panel Consisting of Extracellular Vesicle miRNAs and mRNAs, cfDNA, and CA19-9 Shows Utility for Diagnosis and Staging of Pancreatic Ductal Adenocarcinoma
2. A Novel PiRNA Enhances CA19-9 Sensitivity for Pancreatic Cancer Identification by Liquid Biopsy
3. Analysis of Base-Position Error Rate of Next-Generation Sequencing to Detect Tumor Mutations in Circulating DNA
4. Application of plasma circulating KRAS mutations as a predictive biomarker for targeted treatment of pancreatic cancer
5. Bile-Based Cell-Free DNA Analysis Is a Reliable Diagnostic Tool in Pancreatobiliary Cancer
6. Cell free DNA in patients with pancreatic adenocarcinoma: clinicopathologic correlations
7. Circulating Cell-Free Tumor DNA in Advanced Pancreatic Adenocarcinoma Identifies Patients With Worse Overall Survival
8. Circulating TP53 mutations are associated with early tumor progression and poor survival in pancreatic cancer patients treated with FOLFIRINOX
9. Circulating Tumor DNA as a Sensitive Marker in Patients Undergoing Irreversible Electroporation for Pancreatic Cancer
10. Circulating tumor DNA quantity is related to tumor volume and both predict survival in metastatic pancreatic ductal adenocarcinoma
11. Clinical Utility of Circulating Tumor DNA for Molecular Assessment and Precision Medicine in Pancreatic Cancer
12. Clinical utility of circulating tumor DNA for molecular assessment in pancreatic cancer
13. Combining the amplification refractory mutation system and high-resolution melting analysis for KRAS mutation detection in clinical samples
14. Comprehensive ctDNA Measurements Improve Prediction of Clinical Outcomes and Enable Dynamic Tracking of Disease Progression in Advanced Pancreatic Cancer
15. Correlative Analysis of Tumor-Informed Circulating Tumor DNA (ctDNA) and the Survival Outcomes of Patients with Pancreatic Adenocarcinoma
16. Detection of Circulating Tumor DNA in Patients with Pancreatic Cancer Using Digital Next-Generation Sequencing
17. Detection of KRAS Mutations in Circulating Tumor DNA by Digital PCR in Early Stages of Pancreatic Cancer
18. Diagnostic and Prognostic Values of KRAS Mutations on EUS-FNA Specimens and Circulating Tumor DNA in Patients With Pancreatic Cancer
19. Digital next-generation sequencing of cell-free DNA for pancreatic cancer
20. Efficient and accurate KRAS genotyping using digital PCR combined with melting curve analysis for ctDNA from pancreatic cancer patients
21. Enhancing the compatibility of BioCaRGOS silica sol-gel technology with ctDNA extraction and droplet digital PCR (ddPCR) analysis

22. Extracellular vesicles are the primary source of blood-borne tumour-derived mutant KRAS DNA early in pancreatic cancer
23. Genome-wide profiling of circulating tumor DNA depicts landscape of copy number alterations in pancreatic cancer with liver metastasis
24. KRAS Mutant Allele Fraction in Circulating Cell-Free DNA Correlates With Clinical Stage in Pancreatic Cancer Patients
25. Liquid Biopsy and Automated Next-Generation Sequencing: Achieving Results in 27 Hours Within a Community Setting
26. Monitoring Tumor Burden in Response to FOLFIRINOX Chemotherapy Via Profiling Circulating Cell-Free DNA in Pancreatic Cancer
27. Mutant KRAS Circulating Tumor DNA Is an Accurate Tool for Pancreatic Cancer Monitoring
28. Nanomaterial isolated extracellular vesicles enable high precision identification of tumor biomarkers for pancreatic cancer liquid biopsy
29. Optimization of Sources of Circulating Cell-Free DNA Variability for Downstream Molecular Analysis
30. Parallel Analysis of Pre- and Postoperative Circulating Tumor DNA and Matched Tumor Tissues in Resectable Pancreatic Ductal Adenocarcinoma: A Prospective Cohort Study
31. Plasma Circulating Tumor DNA in Pancreatic Cancer Patients Is a Prognostic Marker
32. Preoperative detection of KRAS G12D mutation in ctDNA is a powerful predictor for early recurrence of resectable PDAC patients
33. Prognostic impact of postoperative circulating tumor DNA as a molecular minimal residual disease marker in patients with pancreatic cancer undergoing surgical resection
34. Reliable on-treatment prognostication and target identification with a customized assay for circulating tumor DNA in patients with newly diagnosed pancreatic cancer
35. Repeated mutKRAS ctDNA measurements represent a novel and promising tool for early response prediction and therapy monitoring in advanced pancreatic cancer
36. Role of preoperative circulating tumor DNA in predicting occult metastases in resectable and borderline resectable pancreatic ductal adenocarcinoma
37. Small RNA sequencing analysis of peptide-affinity isolated plasma extracellular vesicles distinguishes pancreatic cancer patients from non-affected individuals
38. Somatic Mutation Detection in Tumor Tissue and Matched Cell-Free DNA Using PCR-Based Methods in Pancreatic Cancer Patients Undergoing Upfront Resection
39. Tumor-Informed Approach Improved ctDNA Detection Rate in Resected Pancreatic Cancer
40. Ultrasensitive plasma ctDNA KRAS assay for detection, prognosis, and assessment of therapeutic response in patients with unresectable pancreatic ductal adenocarcinoma
41. ctDNA whole exome sequencing in pancreatic ductal adenocarcinoma unveils organ-dependent metastatic mechanisms and identifies actionable alterations in fast progressing patients
42. miR-6855-5p Enhances Radioresistance and Promotes Migration of Pancreatic Cancer by Inducing Epithelial-Mesenchymal Transition via Suppressing FOXA1: Potential of Plasma Exosomal miR-6855-5p as an Indicator of Radiosensitivity in Patients with Pancreatic Cancer

B. Records excluded at title/abstract screening, including duplicate or overlapping records identified during screening (n=36)

1. Combining the amplification refractory mutation system and high-resolution melting analysis for KRAS mutation detection in clinical samples; general KRAS detection/methodology paper; not clearly PDAC plasma ctDNA-focused.
2. Enhancing the compatibility of BioCaRGOS silica sol-gel technology with ctDNA extraction and droplet digital PCR (ddPCR) analysis; analytical methods paper; not a primary PDAC ctDNA study.
3. Extracellular vesicles are the primary source of blood-borne tumour-derived mutant KRAS DNA early in pancreatic cancer; extracellular vesicle focused; outside ctDNA/cfDNA analyte scope.
4. Optimization of Sources of Circulating Cell-Free DNA Variability for Downstream Molecular Analysis; general cfDNA pre-analytical variability/methodology study; not PDAC-specific ctDNA reporting.
5. Clinical Utility of Circulating Tumor DNA for Molecular Assessment and Precision Medicine in Pancreatic Cancer; book chapter/conference proceedings; not an eligible primary journal article.
6. Nanomaterial isolated extracellular vesicles enable high precision identification of tumor biomarkers for pancreatic cancer liquid biopsy; extracellular vesicle biomarker study; not ctDNA/cfDNA-focused.
7. miR-6855-5p Enhances Radioresistance and Promotes Migration of Pancreatic Cancer by Inducing Epithelial-Mesenchymal Transition via Suppressing FOXA1: Potential of Plasma Exosomal miR-6855-5p as an Indicator of Radiosensitivity in Patients with Pancreatic Cancer; exosomal microRNA study; not ctDNA/cfDNA.
8. Small RNA sequencing analysis of peptide-affinity isolated plasma extracellular vesicles distinguishes pancreatic cancer patients from non-affected individuals; extracellular vesicle/small RNA study; not ctDNA/cfDNA.
9. A Novel PiRNA Enhances CA19-9 Sensitivity for Pancreatic Cancer Identification by Liquid Biopsy; piRNA biomarker paper; not ctDNA/cfDNA.
10. Bile-Based Cell-Free DNA Analysis Is a Reliable Diagnostic Tool in Pancreatobiliary Cancer; wrong biospecimen (bile rather than plasma) and mixed disease group.
11. A Multianalyte Panel Consisting of Extracellular Vesicle miRNAs and mRNAs, cfDNA, and CA19-9 Shows Utility for Diagnosis and Staging of Pancreatic Ductal Adenocarcinoma; multianalyte panel; ctDNA/cfDNA was not the principal analyte or reporting framework.
12. Plasma Next Generation Sequencing and Droplet Digital-qPCR-Based Quantification of Circulating Cell-Free RNA for Noninvasive Early Detection of Cancer; cell-free RNA study; not ctDNA/cfDNA.
13. Analysis of Base-Position Error Rate of Next-Generation Sequencing to Detect Tumor Mutations in Circulating DNA; general sequencing error/methods paper; not clearly pancreatic cancer–specific.
14. A meta-analysis of reversion mutations in BRCA genes identifies signatures of DNA end-joining repair mechanisms driving therapy resistance; not pancreatic cancer–specific.
15. Clinical value of ctDNA in upper-GI cancers: A systematic review and meta-analysis; not strictly pancreatic cancer–specific.
16. New cutoff of CA19-9 values for predicting pancreatic cancer using liquid biopsy; not a ctDNA study.
17. Liquid Biopsy for Non-Invasive Monitoring of Tumour Evolution and Response to Therapy; broad review; not pancreas-specific primary evidence.

18. Effect of ctDNA whole-exome sequencing in pancreatic adenocarcinoma on prognostic value, actionable alterations, and organ tropisms in metastatic disease; conference abstract only; corresponding full-text article was considered separately.
19. Plasma Circulating Tumor DNA in Pancreatic Cancer Patients Is a Prognostic Marker; duplicate/overlapping record in the title/abstract audit trail
20. Analysis of base-position error rate of next-generation sequencing to detect tumor mutations in circulating DNA; duplicate record in the title/abstract audit trail.
21. A multianalyte panel consisting of extracellular vesicle miRNAs and mRNAs, cfDNA, and CA19-9 shows utility for diagnosis and staging of pancreatic ductal adenocarcinoma; duplicate/overlapping record in the title/abstract audit trail.
22. Repeated mutKRAS ctDNA measurements represent a novel and promising tool for early response prediction and therapy monitoring in advanced pancreatic cancer; duplicate/overlapping record in the title/abstract audit trail. Considered as full text
23. Preoperative detection of KRAS G12D mutation in ctDNA is a powerful predictor for early recurrence of resectable PDAC patients; - duplicate/overlapping record in the title/abstract audit trail. Considered as full text
24. Correlative Analysis of Tumor-Informed Circulating Tumor DNA (ctDNA) and the Survival Outcomes of Patients with Pancreatic Adenocarcinoma; - duplicate/overlapping record in the title/abstract audit trail. Considered as full text
25. Role of preoperative circulating tumor DNA in predicting occult metastases in resectable and borderline resectable pancreatic ductal adenocarcinoma; - duplicate/overlapping record in the title/abstract audit trail. Considered as full text
26. Reliable on-treatment prognostication and target identification with a customized assay for circulating tumor DNA in patients with newly diagnosed pancreatic cancer; - duplicate/overlapping record in the title/abstract audit trail. Considered as full text
27. Liquid Biopsy and Automated Next-Generation Sequencing: Achieving Results in 27 Hours Within a Community Setting; – Duplicate/overlapping record in the title/abstract audit trail. Later excluded on the basis of full text
28. Monitoring tumor burden in response to FOLFIRINOX chemotherapy via profiling circulating cell-free DNA in pancreatic cancer; duplicate/overlapping record in the title/abstract audit.
29. Plasma Circulating Tumor DNA in Pancreatic Cancer Patients Is a Prognostic Marker – duplicate/overlapping record in the title/abstract audit trail
30. Bile-Based Cell-Free DNA Analysis Is a Reliable Diagnostic Tool in Pancreatobiliary Cancer - duplicate/overlapping record in the title/abstract audit trail
31. Cell free DNA in patients with pancreatic adenocarcinoma: clinicopathologic correlations duplicate/overlapping record in the title/abstract audit trail
32. Circulating Cell-Free Tumor DNA in Advanced Pancreatic Adenocarcinoma Identifies Patients With Worse Overall Survival; duplicate/overlapping record in the title/abstract audit trail
33. Circulating TP53 mutations are associated with early tumor progression and poor survival in pancreatic cancer patients treated with FOLFIRINOX; duplicate/overlapping record in the title/abstract audit trail
34. Detection of Circulating Tumor DNA in Patients with Pancreatic Cancer Using Digital Next-Generation Sequencing; duplicate/overlapping record in the title/abstract audit trail
35. Parallel Analysis of Pre- and Postoperative Circulating Tumor DNA and Matched Tumor Tissues in Resectable Pancreatic Ductal Adenocarcinoma: A Prospective Cohort Study; duplicate/overlapping record in the title/abstract audit trail

36. Tumor-Informed Approach Improved ctDNA Detection Rate in Resected Pancreatic Cancer; duplicate/overlapping record in the title/abstract audit trail

C. Full-text articles assessed for eligibility (including primary studies and systematic reviews/meta-analyses) (n= 44)

1. Somatic Mutation Detection in Tumor Tissue and Matched Cell-Free DNA Using PCR-Based Methods in Pancreatic Cancer Patients Undergoing Upfront Resection
2. Integrated Analysis of Cell-Free DNA and Novel Protein Biomarkers for Stratification and Therapy Monitoring in Stage IV Pancreatic Cancer: A Preliminary Study
3. ctDNA whole exome sequencing in pancreatic ductal adenocarcinoma unveils organ-dependent metastatic mechanisms and identifies actionable alterations in fast progressing patients
4. Application of plasma circulating KRAS mutations as a predictive biomarker for targeted treatment of pancreatic cancer
5. Efficient and accurate KRAS genotyping using digital PCR combined with melting curve analysis for ctDNA from pancreatic cancer patients
6. Prognostic impact of postoperative circulating tumor DNA as a molecular minimal residual disease marker in patients with pancreatic cancer undergoing surgical resection
7. Mutation Analysis of Pancreatic Juice and Plasma for the Detection of Pancreatic Cancer
8. Comprehensive ctDNA Measurements Improve Prediction of Clinical Outcomes and Enable Dynamic Tracking of Disease Progression in Advanced Pancreatic Cancer
9. Diagnostic and Prognostic Values of KRAS Mutations on EUS-FNA Specimens and Circulating Tumor DNA in Patients With Pancreatic Cancer
10. Repeated mutKRAS ctDNA measurements represent a novel and promising tool for early response prediction and therapy monitoring in advanced pancreatic cancer
11. Circulating Tumor DNA as a Sensitive Marker in Patients Undergoing Irreversible Electroporation for Pancreatic Cancer
12. Detection of KRAS Mutations in Circulating Tumor DNA by Digital PCR in Early Stages of Pancreatic Cancer
13. KRAS Mutant Allele Fraction in Circulating Cell-Free DNA Correlates With Clinical Stage in Pancreatic Cancer Patients
14. Circulating tumor DNA quantity is related to tumor volume and both predict survival in metastatic pancreatic ductal adenocarcinoma
15. Tumor-Informed Approach Improved ctDNA Detection Rate in Resected Pancreatic Cancer
16. Parallel Analysis of Pre- and Postoperative Circulating Tumor DNA and Matched Tumor Tissues in Resectable Pancreatic Ductal Adenocarcinoma: A Prospective Cohort Study
17. Circulating Cell-Free Tumor DNA in Advanced Pancreatic Adenocarcinoma Identifies Patients With Worse Overall Survival
18. Circulating TP53 mutations are associated with early tumor progression and poor survival in pancreatic cancer patients treated with FOLFIRINOX
19. Detection of Circulating Tumor DNA in Patients with Pancreatic Cancer Using Digital Next-Generation Sequencing
20. Preoperative detection of KRAS G12D mutation in ctDNA is a powerful predictor for early recurrence of resectable PDAC patients

21. Monitoring Tumor Burden in Response to FOLFIRINOX Chemotherapy Via Profiling Circulating Cell-Free DNA in Pancreatic Cancer
22. Ultrasensitive plasma ctDNA KRAS assay for detection, prognosis, and assessment of therapeutic response in patients with unresectable pancreatic ductal adenocarcinoma
23. Clinical utility of circulating tumor DNA for molecular assessment in pancreatic cancer
24. Digital next-generation sequencing of cell-free DNA for pancreatic cancer
25. Genome-wide profiling of circulating tumor DNA depicts landscape of copy number alterations in pancreatic cancer with liver metastasis
26. Mutant KRAS Circulating Tumor DNA Is an Accurate Tool for Pancreatic Cancer Monitoring
27. Plasma circulating tumor DNA in pancreatic cancer patients is a prognostic marker
28. Role of preoperative circulating tumor DNA in predicting occult metastases in resectable and borderline resectable pancreatic ductal adenocarcinoma
29. Correlative analysis of tumor-informed circulating tumor DNA (ctDNA) and the survival outcomes of patients with pancreatic adenocarcinoma
30. Reliable on-treatment prognostication and target identification with a customized assay for circulating tumor DNA in patients with newly diagnosed pancreatic cancer
31. Cell free DNA in patients with pancreatic adenocarcinoma: clinicopathologic correlations
32. Liquid Biopsy and Automated Next-Generation Sequencing: Achieving Results in 27 Hours Within a Community Setting
33. Circulating tumour DNA (ctDNA) as a predictor of progression-free and overall survival in non-resectable pancreatic cancer: a systematic review and meta-analysis
34. The role of novel biomarkers in the early diagnosis of pancreatic cancer: A systematic review and meta-analysis
35. Prognostic utility of preoperative and postoperative KRAS-mutated circulating tumor DNA (ctDNA) in resected pancreatic ductal adenocarcinoma: A systematic review and meta-analysis
36. Liquid biopsy after resection of pancreatic adenocarcinoma and its relation to oncological outcomes. Systematic review and meta-analysis
37. Prognostic role of cell-free DNA biomarkers in pancreatic adenocarcinoma: A systematic review and meta-analysis
38. A systematic review and meta-analysis of the association between circulating tumor DNA (ctDNA) and prognosis in pancreatic cancer
39. Meta-Analysis of Circulating Cell-Free DNA's Role in the Prognosis of Pancreatic Cancer
40. Prognostic value of circulating tumor DNA in pancreatic cancer: a systematic review and meta-analysis
41. Diagnostic value of various liquid biopsy methods for pancreatic cancer: A systematic review and meta-analysis
42. Circulating tumor DNA as a prognostic indicator in resectable pancreatic ductal adenocarcinoma: A systematic review and meta-analysis
43. The Role of Circulating Tumor DNA in Surgical Management of Pancreatic Cancer: Systematic Review and Meta-analysis
44. Prognostic value of circulating cell-free DNA in patients with pancreatic cancer: A systemic review and meta-analysis

D. Records excluded after full text review

1. Liquid Biopsy and Automated Next-Generation Sequencing: Achieving Results in 27 Hours Within a Community Setting; case report; not eligible as a primary cohort study.

E. Records included after manual searching (n=5)

1. Detection of K-ras gene mutation by liquid biopsy in patients with pancreatic cancer- Kinugasa et al
2. Prognostic value of circulating tumour DNA in patients undergoing curative resection for pancreatic cancer – Hadano et al
3. Clinical relevance of circulating KRAS mutated DNA in plasma from patients with advanced pancreatic cancer – Tjevnsvoll et al
4. Targeted deep sequencing of circulating tumor DNA in metastatic pancreatic cancer – Berger et al
5. Circulating Tumor DNA Is an Accurate Diagnostic Tool and Strong Prognostic Marker in Pancreatic Cancer. – Sellahewa et al

F. Final Records included (36 Primary ctDNA studies)

1. Brychta, N., T. Krahn, and O. von Ahsen, Detection of KRAS mutations in circulating tumor DNA by digital PCR in early stages of pancreatic cancer. Clinical chemistry, 2016. 62(11): p. 1482–1491.
2. Chen, I., et al., Ultrasensitive plasma ctDNA KRAS assay for detection, prognosis, and assessment of therapeutic response in patients with unresectable pancreatic ductal adenocarcinoma. Oncotarget, 2017. 8(58): p. 97769.
3. Guo, S., et al., Preoperative detection of KRAS G12D mutation in ctDNA is a powerful predictor for early recurrence of resectable PDAC patients. British journal of cancer, 2020. 122(6): p. 857–867.
4. Macgregor-Das, A., et al., Detection of circulating tumor DNA in patients with pancreatic cancer using digital next-generation sequencing. The Journal of Molecular Diagnostics, 2020. 22(6): p. 748–756.
5. van der Sijde, F., et al., Circulating TP53 mutations are associated with early tumor progression and poor survival in pancreatic cancer patients treated with FOLFIRINOX. Therapeutic Advances in Medical Oncology, 2021. 13: p. 17588359211033704.
6. Wang, R., et al., Diagnostic and prognostic values of KRAS mutations on EUS-FNA specimens and circulating tumor DNA in patients with pancreatic cancer. Clinical and Translational Gastroenterology, 2022. 13(5): p. e00487.
7. Watanabe, K., et al., Tumor-informed approach improved ctDNA detection rate in resected pancreatic cancer. International journal of molecular sciences, 2022. 23(19): p. 11521.
8. Hata, T., et al., Prognostic impact of postoperative circulating tumor DNA as a molecular minimal residual disease marker in patients with pancreatic cancer undergoing surgical resection. Journal of Hepato-Biliary-Pancreatic Sciences, 2023. 30(6): p. 815–824.
9. Levink, I.J., et al., Mutation analysis of pancreatic juice and plasma for the detection of pancreatic cancer. International Journal of Molecular Sciences, 2023. 24(17): p. 13116.
10. Lee, M.R., et al., Application of plasma circulating KRAS mutations as a predictive biomarker for targeted treatment of pancreatic cancer. Cancer Science, 2024. 115(4): p. 1283–1295.

11. Murakami, T., et al., Role of preoperative circulating tumor DNA in predicting occult metastases in resectable and borderline resectable pancreatic ductal adenocarcinoma. World Journal of Gastroenterology, 2025. 31(32): p. 109383.

12. Zhang, Y., et al., Correlative analysis of tumor-Informed Circulating tumor DNA (ctDNA) and the survival outcomes of patients with pancreatic adenocarcinoma. Biomedicines, 2025. 13(5): p. 1124.

13. Kinugasa, H., et al., Detection of K-ras gene mutation by liquid biopsy in patients with pancreatic cancer. Cancer, 2015. 121(13): p. 2271–2280.

14. Takai, E., et al., Clinical utility of circulating tumor DNA for molecular assessment in pancreatic cancer. Scientific reports, 2015. 5(1): p. 18425.

15. Tjensvoll, K., et al., Clinical relevance of circulating KRAS mutated DNA in plasma from patients with advanced pancreatic cancer. Molecular oncology, 2016. 10(4): p. 635–643.

16. Berger, A.W., et al., Targeted deep sequencing of circulating tumor DNA in metastatic pancreatic cancer. Oncotarget, 2017. 9(2): p. 2076.

17. Pietrasz, D., et al., Plasma circulating tumor DNA in pancreatic cancer patients is a prognostic marker. Clinical Cancer Research, 2017. 23(1): p. 116–123.

18. Kruger, S., et al., Repeated mutKRAS ctDNA measurements represent a novel and promising tool for early response prediction and therapy monitoring in advanced pancreatic cancer. Annals of Oncology, 2018. 29(12): p. 2348–2355.

19. Perets, R., et al., Mutant KRAS circulating tumor DNA is an accurate tool for pancreatic cancer monitoring. The oncologist, 2018. 23(5): p. 566–572.

20. Wang, Z.-Y., et al., KRAS mutant allele fraction in circulating cell-free DNA correlates with clinical stage in pancreatic cancer patients. Frontiers in oncology, 2019. 9: p. 1295.

21. Wei, T., et al., Monitoring tumor burden in response to FOLFIRINOX chemotherapy via profiling circulating cell-free DNA in pancreatic cancer. Molecular cancer therapeutics, 2019. 18(1): p. 196–203.

22. Strijker, M., et al., Circulating tumor DNA quantity is related to tumor volume and both predict survival in metastatic pancreatic ductal adenocarcinoma. International journal of cancer, 2020. 146(5): p. 1445–1456.

23. Wei, T., et al., Genome-wide profiling of circulating tumor DNA depicts landscape of copy number alterations in pancreatic cancer with liver metastasis. Molecular Oncology, 2020. 14(9): p. 1966–1977.

24. Takano, S., et al., Digital next-generation sequencing of cell-free DNA for pancreatic cancer. JGH Open, 2021. 5(4): p. 508–516.

25. Botrus, G., et al., Circulating cell-free tumor DNA in advanced pancreatic adenocarcinoma identifies patients with worse overall survival. Frontiers in oncology, 2022. 11: p. 794009.

26. Lee, J.-S., et al., Parallel analysis of pre-and postoperative circulating tumor DNA and matched tumor tissues in resectable pancreatic ductal adenocarcinoma: a prospective cohort study. Clinical Chemistry, 2022. 68(12): p. 1509–1518.

27. Lapin, M., et al., Comprehensive ctDNA measurements improve prediction of clinical outcomes and enable dynamic tracking of disease progression in advanced pancreatic cancer. Clinical Cancer Research, 2023. 29(7): p. 1267–1278.

28. Tanaka, J., et al., Efficient and accurate KRAS genotyping using digital PCR combined with melting curve analysis for ctDNA from pancreatic cancer patients. Scientific Reports, 2023. 13(1): p. 3039.

29. Huerta, M., et al., ctDNA whole exome sequencing in pancreatic ductal adenocarcinoma unveils organ-dependent metastatic mechanisms and identifies actionable alterations in fast progressing patients. Translational Research, 2024. 271: p. 105–115.

30. Theparee, T., et al., Cell free DNA in patients with pancreatic adenocarcinoma: clinicopathologic correlations. Scientific Reports, 2024. 14(1): p. 15744.

31. Petersson, A., et al., Reliable on-treatment prognostication and target identification with a customized assay for circulating tumor DNA in patients with newly diagnosed pancreatic cancer. Scientific Reports, 2025. 15(1): p. 34481.

32. Zavrtanik Čarni, H., et al., Somatic mutation detection in tumor tissue and matched Cell-Free DNA using PCR-Based methods in pancreatic cancer patients undergoing upfront resection. International journal of molecular sciences, 2025. 26(17): p. 8518.

33. Hadano, N., et al., Prognostic value of circulating tumour DNA in patients undergoing curative resection for pancreatic cancer. British journal of cancer, 2016. 115(1): p. 59–65.

34. Lin, M., et al., Circulating tumor DNA as a sensitive marker in patients undergoing irreversible electroporation for pancreatic cancer. Cellular Physiology and Biochemistry, 2018. 47(4): p. 1556–1564.

35. Hussung, S., et al., Integrated analysis of cell-free DNA and novel protein biomarkers for stratification and therapy monitoring in stage IV pancreatic cancer: a preliminary study. Diagnostics, 2024. 15(1): p. 49.

36. Sellahewa, R., et al., Circulating tumor DNA is an accurate diagnostic tool and strong prognostic marker in pancreatic cancer. Pancreas, 2023. 52(3): e. 188-195.

G. Final records included (12 Meta-analysis studies)

1. Steiniche, M.M.; Callesen, L.B.; Vlk, E.H.; Ventzel, L.; Timm, S.; Andersen, R.F.; Lindgaard, S.C.; Hansen, T.F.; Ladekarl, M.; Spindler, K.-L.G. Circulating tumour DNA (ctDNA) as a predictor of progression-free and overall survival in non-resectable pancreatic cancer: a systematic review and meta-analysis. The Journal of Liquid Biopsy 2025, 100441.

2. Zheng, Z.; Lu, Z.; Yan, F.; Song, Y. The role of novel biomarkers in the early diagnosis of pancreatic cancer: A systematic review and meta-analysis. PLoS One 2025, 20, e0322720.

3. Alqahtani, A.; Alloghbi, A.; Coffin, P.; Yin, C.; Mukherji, R.; Weinberg, B.A. Prognostic utility of preoperative and postoperative KRAS-mutated circulating tumor DNA (ctDNA) in resected pancreatic ductal adenocarcinoma: a systematic review and meta-analysis. Surgical Oncology 2023, 51, 102007.

4. Vidal, L.; Pando, E.; Blanco, L.; Fabregat-Franco, C.; Castet, F.; Sierra, A.; Macarulla, T.; Balsells, J.; Charco, R.; Vivancos, A. Liquid biopsy after resection of pancreatic adenocarcinoma and its relation to oncological outcomes. Systematic review and meta-analysis. Cancer Treatment Reviews 2023, 120, 102604.

5. Bunduc, S.; Gede, N.; Vancsa, S.; Lillik, V.; Kiss, S.; Dembrovszky, F.; Eross, B.; Szakacs, Z.; Gheorghe, C.; Miko, A. Prognostic role of cell-free DNA biomarkers in pancreatic adenocarcinoma: A systematic review and meta–analysis. Critical Reviews in Oncology/Hematology 2022, 169, 103548.

6. Guven, D.C.; Sahin, T.K.; Yildirim, H.C.; Aktepe, O.H.; Dizdar, O.; Yalcin, S. A systematic review and meta-analysis of the association between circulating tumor DNA (ctDNA) and prognosis in pancreatic cancer. Critical reviews in oncology/hematology 2021, 168, 103528.

7. Milin-Lazovic, J.; Madzarevic, P.; Rajovic, N.; Djordjevic, V.; Milic, N.; Pavlovic, S.; Veljkovic, N.; Milic, N.M.; Radenkovic, D. Meta-analysis of circulating cell-free DNA's role in the prognosis of pancreatic cancer. Cancers 2021, 13, 3378.
8. Fang, Z.; Meng, Q.; Zhang, B.; Shi, S.; Liu, J.; Liang, C.; Hua, J.; Yu, X.; Xu, J.; Wang, W. Prognostic value of circulating tumor DNA in pancreatic cancer: a systematic review and meta-analysis. Aging (Albany NY) 2020, 13, 2031.
9. Zhu, Y.; Zhang, H.; Chen, N.; Hao, J.; Jin, H.; Ma, X. Diagnostic value of various liquid biopsy methods for pancreatic cancer: A systematic review and meta-analysis. Medicine 2020, 99, e18581.
10. Lee, J.-S.; Rhee, T.-M.; Pietrasz, D.; Bachet, J.-B.; Laurent-Puig, P.; Kong, S.-Y.; Takai, E.; Yachida, S.; Shibata, T.; Lee, J.W. Circulating tumor DNA as a prognostic indicator in resectable pancreatic ductal adenocarcinoma: A systematic review and meta-analysis. Scientific Reports 2019, 9, 16971.
11. Borges, F.C.; Pinto, M.S.; Borges, M.F.; João, A.A.; Francisco, E.; Sousa, M.; Aral, M.; Oliveira, V.; Cunha, J.F.; Mehrabi, A. The Role of Circulating Tumor DNA in Surgical Management of Pancreatic Cancer: Systematic Review and Meta-analysis. Annals of Surgery 2026, 10.1097.
12. Chen, L.; Zhang, Y.; Cheng, Y.; Zhang, D.; Zhu, S.; Ma, X. Prognostic value of circulating cell-free DNA in patients with pancreatic cancer: A systemic review and meta-analysis. Gene 2018, 679, 328–334.
